# Comprehensive analysis of the NAC transcription factor gene family in *Kandelia obovata* reveals potential members related to chilling tolerance

**DOI:** 10.3389/fpls.2022.1048822

**Published:** 2022-11-17

**Authors:** Zhaokui Du, Shixian You, Dang Yang, Yutian Tao, Yunxiao Zhu, Wen Sun, Zhengman Chen, Junmin Li

**Affiliations:** ^1^ Zhejiang Provincial Key Laboratory of Plant Evolutionary Ecology and Conservation, Taizhou University, Taizhou, China; ^2^ Section of Maritime Space and Island Management, Yuhuan Municipal Bureau of Natural Resources and Planning, Yuhuan, China; ^3^ Department of Security Production Management, Taizhou Circular Economy Development Co., Ltd., Taizhou, China

**Keywords:** chilling stress, expression profiles, gene duplication, *Kandelia obovata*, *NAC* gene family, phylogenetic analysis

## Abstract

**Background:**

*Kandelia obovata* is an important mangrove species extensively distributed in Eastern Asia that is susceptible to low-temperature stress. NAC (NAM, ATAF1/2 and CUC2) domain proteins are transcription factors (TFs) that play various roles in plant growth and development and in the plant response to environmental stresses. Nevertheless, genome-wide analyses of *K. obovata NAC* genes (*KoNACs*) and their responses to chilling stress have rarely been studied.

**Methods:**

The *KoNAC* gene family was identified and characterized using bioinformatic analysis, the subcellular location of some NAC proteins was confirmed using confocal microscopy analysis, and the *KoNACs* that responded to chilling stress were screened using RNA-seq and qRT-PCR analysis.

**Results:**

A total of 79 *KoNACs* were identified, and they were unequally distributed across all 18 chromosomes of *K. obovata*. The KoNAC proteins could be divided into 16 subgroups according to the phylogenetic tree based on NAC family members of *Arabidopsis thaliana*. The *KoNACs* exhibited greater synteny with *A. thaliana* sequences than with *Oryza sativa* sequences, indicating that *KoNACs* underwent extensive evolution after the divergence of dicotyledons and monocotyledons. Segmental duplication was the main driving force of the expansions of *KoNAC* genes. Confocal microscopy analysis verified that the four randomly selected KoNACs localized to the nucleus, indicating the accuracy of the bioinformatic predictions. Tissue expression pattern analysis demonstrated that some *KoNAC* genes showed tissue-specific expression, suggesting that these *KoNACs* might be important for plant development and growth. Additionally, the expression levels of 19 *KoNACs* were significantly (15 positively and 4 negatively) induced by cold treatment, demonstrating that these *KoNACs* might play important roles during cold stress responses and might be candidate genes for the genetic engineering of *K. obovata* with enhanced chilling stress tolerance. Coexpression network analysis revealed that 381 coexpressed pairs (between 13 *KoNACs* and 284 other genes) were significantly correlated.

**Conclusions:**

Seventy-nine *KoNACs* were identified in *K. obovata*, nineteen of which displayed chilling-induced expression patterns. These genes may serve as candidates for functional analyses of *KoNACs* engaged in chilling stress. Our results lay the foundation for evolutionary analyses of *KoNACs* and their molecular mechanisms in response to environmental stress.

## Introduction

Mangrove forests are composed of woody plants that grow in subtropical and tropical coastal intertidal zones, where they play an important role in preventing wind, attenuating waves, purifying seawater, maintaining biodiversity, supplying seafood, and sequestering carbon (Trégarot et al., 2021). Mangroves grow in harsh conditions involving anaerobic soils, extreme tides, high salinity, strong winds, sea-level rise, and extreme climate events (Guo et al., 2017). Cold stress is one of the most crucial factors limiting the development, growth, and geographical distribution of mangrove plants among all of these abiotic stresses (Shen et al., 2021). Cold stress (low temperature) can be categorized into chilling stress (0−15°C) or freezing stress (< 0°C) according to its intensity (Jiang et al., 2021). Due to their inability to adapt to low temperatures, tropical and subtropical plants typically exhibit sensitivity to cold stress and are susceptible to chilling stress (Wu et al., 2019). On the Pacific coast of North America, the 50% mortality temperature threshold for black mangroves is −0.92°C (Bardou et al., 2020). In early 2008, a chilling event occurred in southern China, resulting in the withering and even death of many mangrove plants (Chen et al., 2017). Recently, a study of mangrove responses to chilling stress reported that the leaves of *Bruguiera gymnorhiza* showed obvious yellowing, curling, and wilting following exposure to a temperature of 5°C for 13 days (Wang et al., 2022).

*Kandelia obovata* is a species of the Rhizophoraceae family that was initially named *K. candel* in the regions of China and Japan and has now been reclassified as a new species (Sheue et al., 2003). As the dominant mangrove species in Eastern Asia, *K. obovata* provides significant ecological functions and valuable socioeconomic services to humans, such as providing habitat, preserving species, safeguarding shorelines, purifying water, and sequestrating carbon (Wu et al., 2020; Wei et al., 2022). *K. obovata* is the most cold-resistant species among mangrove plants, and the northernmost point of its natural distribution is Fuding (27° 20’ N) in Fujian Province, China (Chen et al., 2017). Recently, certain mangrove species have even expanded to higher latitudes due to climate change (Osland et al., 2013). *K. obovata* was introduced to Yueqing Bay (28° 20’ N), Zhejiang Province, where the propagules of adult trees can settle naturally and grow into seedlings (Lu et al., 2022). However, extreme cold climatic events exert a destructive influence on mangroves. For example, a severe chilling injury in the winter of 2015 led to different levels of defoliation and death of *K. obovata* in Yueqing Bay (Lu et al., 2022).

*K. obovata* has a remarkable capability to cope with environmental cold stress. In recent years, an increasing number of research studies have uncovered the physiological and molecular mechanisms by which *K. obovata* adapts to low temperature (Fei et al., 2015; Peng et al., 2015; Su et al., 2019; Fei et al., 2021a; Fei et al., 2021b). An AP2/EREBP transcription factor (TF), KcCBF3, was cloned in our previous study, and it was shown to be involved in the adaptation of *K*. *obovata* to 4°C chilling stress (Du and Li, 2019), which is in accordance with the results of Peng et al. (2020). We also found that 9 WRKY transcription factors (TFs) exhibited 4°C-induced expression patterns, implying that they may play important roles in the response of *K. obovata* to chilling (Du et al., 2022). TFs are proteins that can either activate or suppress the expression of target genes by binding to specific *cis*-elements in promoter regions or interacting with other regulatory factors (Manna et al., 2021). In the past few decades, a large number of research efforts have focused on identifying and characterizing various TFs that participate in plant stress responses in either the ABA-independent pathway or the ABA-dependent pathway, such as WRKY, AP2/EREBP, MYB, bHLH, bZIP, and NAC TFs (Wang et al., 2016).

The acronym NAC originated from the names of three genes that were initially discovered to contain a particular NAC domain: NAM (No Apical Meristem), ATAF1/ATAF2 (Arabidopsis Transcription Activation Factor 1/2), and CUC2 (Cup-shaped Cotyledon 2) (Nakashima et al., 2012). A classic NAC protein consists of a highly conserved DNA-binding NAC domain within the N-terminal region and a variable transcriptional regulatory region at the C-terminal (Shao et al., 2015). The NAC domain is a highly conserved DNA-binding domain containing approximately 150–160 amino acid residues and can be classified into five subdomains (A to E) (Ooka et al., 2003). The function of the NAC domain is relevant to DNA binding, nuclear localization, and the constitution of homodimers as well as heterodimers with other NAC domain-containing TFs (Olsen et al., 2005). In contrast, the C-terminal region of NAC proteins is highly divergent and functions as a transcriptional activator or repressor to regulate the transcription of target genes (Nakashima et al., 2012).

NAC proteins can be found in a variety of plant species and are one of the largest families of TFs in plants. The completion of high-quality plant genome sequences has provided the possibility for the genome-wide analysis of all members belonging to specific gene families (Nuruzzaman et al., 2010). Whole-genome-based studies have revealed 117 *NAC* genes in *Arabidopsis thaliana*, 151 in *Oryza sativa*, 74 in *Vitis vinifera*, 110 in *Solanum tuberosum*, 204 in *Brassica rapa*, and 152 in *Zea mays*, *Glycine max* and *Nicotiana tabacum* thus far (Shao et al., 2015). NACs are plant-specific proteins that play various roles in plant development, morphogenesis, and senescence (Singh et al., 2021). In addition, NAC proteins have been demonstrated to have multiple functions in the responses to biotic stresses, such as bacterial and virus infection (Nakashima et al., 2007), and abiotic stresses, such as drought, salinity, heat, cold, and waterlogging (Nuruzzaman et al., 2013). It was shown that NAC TFs regulate plant chilling resistance through CBF/DREB-dependent or CBF/DREB-independent pathways (Diao et al., 2020). In CBF/DREB-dependent pathway, NACs improve plant cold resistance by inducing the expression of CBF/DREB transcription factors and thus up-regulating the expression of *COR* (cold responsive) genes (Jin et al., 2017; Yong et al., 2019). However, a few NACs increase the plant resistance to cold stress by enhancing the hypersensitivity to ABA in CBF/DREB-independent pathways (Yong et al., 2019); recent study has shown that NAC can also activate the transcription of ethylene synthesis-related enzyme genes by binding to their promoters to induce ethylene synthesis and thus improve plant cold tolerance (Dong et al., 2022).

*K. obovata* widely distributes along the southeastern coast of China and can endure harsh conditions such as high salinity, submergence, hypoxia, or even extremely low temperatures (Fei et al., 2021b; Nizam et al., 2022). Although the important functions of NACs under abiotic stress have been widely illustrated in many plant species, information on these proteins in *K. obovata* is still unavailable. The genome of *K. obovata* was recently published, providing a valuable genomic resource for molecular research on this species (Hu et al., 2020). In the present study, we identified and characterized *NAC* genes (*KoNACs*) in the whole genome of *K. obovata*, surveyed their expression patterns in various tissues, and revealed putative candidate *KoNAC* genes under chilling stress. This study might lay a foundation for further research on the molecular mechanism of *KoNAC* genes in the adaptation of *K. obovata* to chilling stress.

## Materials and methods

### Identification of *KoNAC* genes in *K obovata*

The complete genome data of *K. obovata* were retrieved from the Genome Sequence Archive database (https://bigd.big.ac.cn/gsa/browse/CRA002395). The *NAC* gene sequences of *A. thaliana* and rice were searched and downloaded from the *Arabidopsis* Information Resource (TAIR) website (http://www.Arabidopsis.org) and the Rice Genome Annotation Project (http://rice.plantbiology.msu.edu/), respectively. A hidden Markov model (HMM) profile of the Pfam NAC domain (PF01849) and NAM domain (PF02365) downloaded from the Pfam database (http://pfam.sanger.ac.uk/) was employed to identify *KoNAC* genes by using HMMER 3.2.1 with the default parameters, and the cutoff value was set to 0.01. The putative KoNACs were further validated by screening for NAC and NAM domains in the Pfam database (http://pfam.xfam.org/) and were finally confirmed as the KoNAC proteins after removing redundant sequences with CD-HIT program (http://cd-hit.org/) and performing BLASTP searches. The physicochemical properties of the KoNAC proteins, including their molecular weight (MW) and isoelectric point (pI), were analyzed with the online software ExPASy (https://www.expasy.org). The subcellular localization of the KoNAC proteins was determined with the PSORT program (https://psort.hgc.jp/).

### Phylogenetic analysis of KoNAC proteins

The NAC protein sequences of *A. thaliana* were obtained from the TAIR database (http://www.Arabidopsis.org). An unrooted phylogenetic tree consisting of KoNACs and NACs from *A. thaliana* was constructed with software MEGA6.0 using the neighbor-joining (NJ) method with 1,000 bootstrap iterations. All KoNACs were categorized into different subgroups based on the classification of NAC proteins from *A. thaliana* (Ooka et al., 2003).

### Conserved motif and gene structure analysis

The conserved motif in the KoNAC protein sequences was searched using the Multiple Expectation Maximization for Motif Elicitation (MEME) program (http://meme-suite.org/tools/meme) with the parameters according to Song et al. (2020). The exon/intron structure pattern of the *KoNAC* genes was analyzed using the Gene Structure Display Server (GSDS) program (http://gsds.cbi.pku.edu.cn/) by comparing their predicted coding sequences with the corresponding full-length genomic DNA sequences. The conserved motifs and exon/intron structure were visualized using TBtools (Chen et al., 2020).

### Chromosomal location, gene duplication and synteny analysis of *KoNAC* genes

The chromosomal localizations of all the *KoNAC* genes were determined from the genome annotation file and mapped using MapChart (Voorrips, 2002). The genome sequence and annotation information of *A. thaliana* and rice were downloaded from TAIR and Ensembl (http://plants.ensembl.org/index.html), respectively. The identification of tandem duplication and segmental duplication was detected by the MCScanX program (Wang et al., 2012) with the default parameters, in short, BLASTP alignment significance: E-value threshold = 10^-5^, MatchScore = 50, GapPenalty = -1, maximum number of intervening genes ≤ 25. The synonymous (Ks) and nonsynonymous (Ka) substitution rates of *KoNAC*-homologous genes were calculated using the online tool available at http://services.cbu.uib.no/tools/kaks, and the selection pressure endured by gene pairs was calculated according to the Ka/Ks ratio.

### *Cis*-Acting Regulatory Element (CARE) analysis

The 2-kb upstream sequences of the transcription start site of the *KoNAC* genes were extracted and submitted to the PlantCARE online server (http://bioinformatics.psb.ugent.be/webtools/plantcare/html/) to predict the putative CAREs engaged in plant development, phytohormone, and abiotic stress responses. The frequency of each CARE in *KoNAC* genes was counted, and the 20 most common CAREs in *KoNAC* genes were visualized using TBtools (Chen et al., 2020).

### Experimental determination of the subcellular localization of several KoNACs

To experimentally analyze the subcellular localization of KoNACs, the cDNAs of four randomly selected *KoNACs* (*KoNAC15*, *KoNAC27*, *KoNAC54*, and *KoNAC58*) were cloned into the expression vector CAM-turbo-GFP-FLAG and fused in frame with a GFP or mCherry gene. The primers used for subcellular localization are listed in **Supplementary Table S1**. The recombinant vector and a mock vector were introduced into *A. thaliana* leaf protoplasts, which were then imaged with a laser scanning confocal microscope (Olympus FV1000 viewer, Tokyo, Japan). *Arabidopsis* leaf protoplast isolation and PEG-mediated transformation were performed as previously described (Yoo et al., 2007).

### Expression profiles of *KoNAC* genes in different tissues and in response to cold stress based on transcriptome data

Transcriptome data of *K. obovata* leaves in response to cold stress were downloaded from the National Center for Biotechnology Information (NCBI) database (Accession number: PRJNA678025). Transcriptome data of different tissues of *K. obovata* were also downloaded from the NCBI database (Accession number: PRJNA416402). In addition to the root, stem, leaf, and fruit tissues, the expression levels of the *KoNAC* genes were investigated in flower, pistil, stamen, sepal, and fruit. The relative gene expression values were expressed as transcripts per million (TPM), and all of the transcriptome data were converted into log_2_ (TPM + 1) values (Zeng et al., 2020). For expression analysis of genes in response to cold stress, the differentially expressed *KoNACs* were chosen based on the criteria of | log_2_ (fold-change) ≥1 | and *P* < 0.05. The expression levels of the *KoNAC* genes were visualized in a heatmap using TBtools software (Chen et al., 2020).

### Plant material and treatments

Mature and pest-free propagules of *K. obovata* were picked from Maoyan Island (28°13′N, 121°10′E), Yuhuan, Zhejiang Province, China. They were washed and grown in river sand in a growth chamber at a temperature of 25°C, 75% humidity, and a photoperiod of 14 h light/10 h dark. The seedlings were watered once a week with Hoagland solution, and they were exposed to 4°C chilling stress for 0, 1, 3, or 12 hours at the six-leaf stage. Then, the leaves were collected and immediately frozen in liquid nitrogen and stored at -80°C in an ultralow temperature freezer for qRT-PCR. All treatments were designed with three independent biological replications.

### Validation of *KoNAC* gene expression levels *via* qRT-PCR

Total RNA was extracted from frozen leaves using an RNASimple Total RNA Kit (Tiangen, Beijing, China) and then used for cDNA synthesis with a TIANScript cDNA Kit (Tiangen, Beijing, China). The concentration of cDNA was determined with a NanoDrop 1000 spectrophotometer (Thermo Scientific, Wilmington, DE, USA). Then, qRT-PCR was carried out on a CFX96 Touch real-time PCR Detection System (Bio-Rad Laboratories, Hercules, CA, USA) with SuperReal PreMix Plus (SYBR Green) (Tiangen, Beijing, China). The primers used for qRT-PCR are shown in **Supplementary Table S2**. The relative gene expression levels of selected *KoNACs* were determined with the 2^-ΔΔCT^ method (Livak and Schmittgen, 2001) using *18S rRNA* of *K. obovata* as the internal reference gene. The data are presented as the mean ± standard error; statistical significance was assessed by one-way ANOVA followed by Duncan’s multiple range tests at *P* < 0.05.

### Prediction of coexpression networks among *KoNAC* genes and potential regulated genes

To further explore the potential activators or repressors associated with *KoNACs*, the Pearson correlation coefficients (PCCs) between *KoNACs* and non-*KoNAC* genes were calculated based on their expression levels using the R package. The absolute value of the PCC ≥ 0.95 and *P value* < 0.001 was set as the screening cutoff, and genes that met this criterion were regarded as potential correlated regulators. Cytoscape software (version 3.6.1) was used to visualize the network.

## Results

### Identification and characterization of *KoNAC* genes

Members of the *KoNAC* family were identified in the *K. obovata* genome *via* HMM searches with profiles PF01849 and PF02365, which represent the NAC and NAM domains, respectively. A total of 79 candidate genes were further identified by local BLASTP searches and were designated *KoNAC1* to *KoNAC79* based on their order on the chromosomes (**Supplementary Table S3**). Basic information of the 79 *KoNAC* genes, including the length of their CDSs and the lengths, MWs, pIs and subcellular localizations of their encoded proteins, was analyzed in the present study. The *KoNAC* CDS lengths ranged from 408 (*KoNAC29*) to 1,941 bp (*KoNAC18*), with an average length of 1,021 bp. The MWs ranged from 15.53 kDa (KoNAC29) to 73.58 kDa (KoNAC18), with an average of 44.58 kDa. The theoretical pIs varied from 4.46 (KoNAC53) to 9.63 (KoNAC11). The subcellular localizations of the 79 KoNAC proteins were predicted to be the nucleus (64), cytoplasm (7), chloroplast (4), mitochondrion (2), vacuole (1) and extracellular space (1).

### Phylogenetic analysis and classification of KoNAC proteins

**Figure 1:**
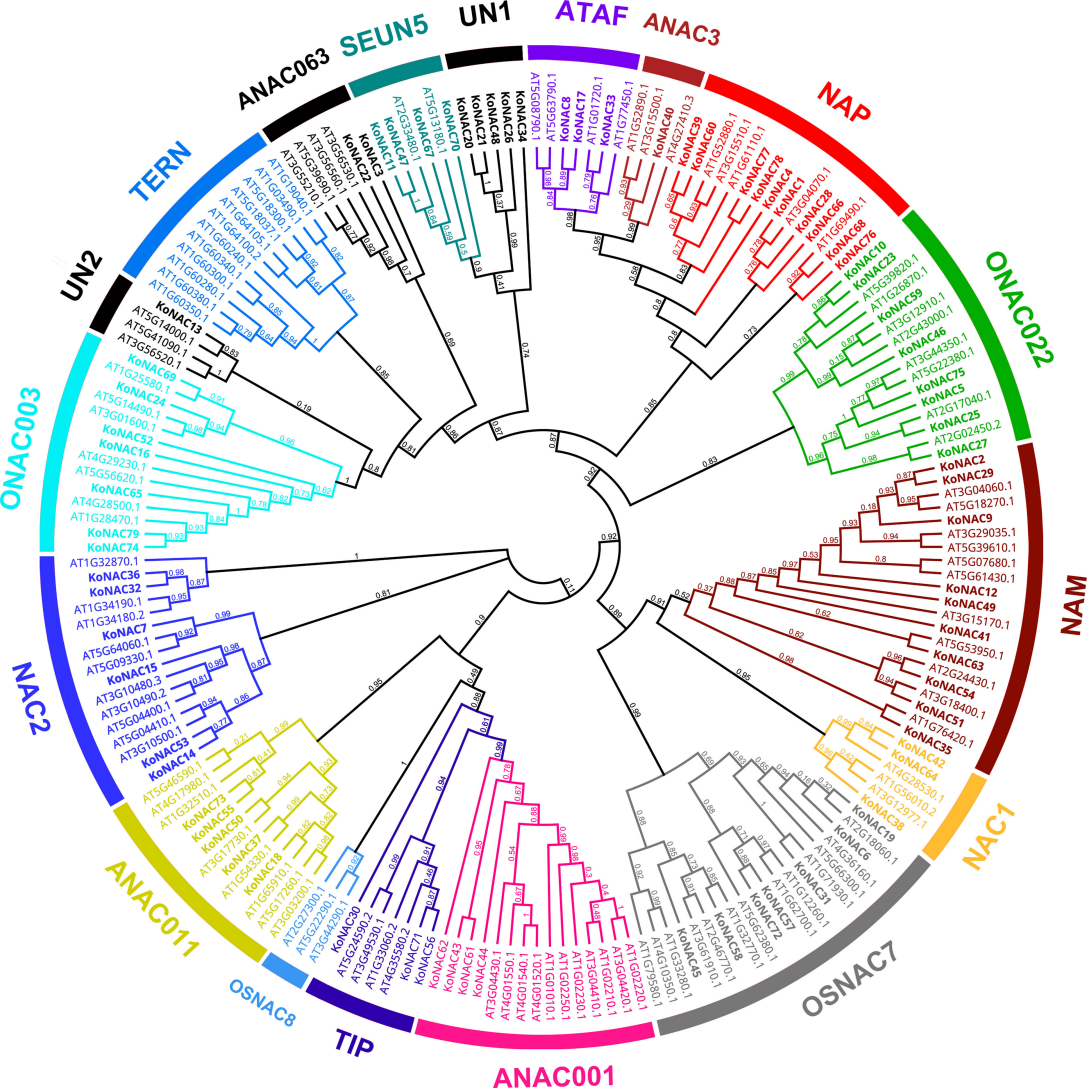
Phylogenetic relationships among NAC proteins identified in *Kandelia obovata* and *Arabidopsis thaliana*. The neighbor-joining (NJ) unrooted phylogenetic tree was constructed according to the NAC protein sequences of *K. obovata* and *A. thaliana* using the program MEGA 6.0. The subfamilies are labeled and indicated with different colors, and the unclassified KoNACs are represented by the abbreviation “UN”.

To explore the evolutionary relationships of the KoNAC members, an unrooted phylogenetic tree was constructed by using the protein sequences of KoNACs and NACs from *A. thaliana*. The results showed that the 79 KoNAC proteins could be divided into 16 subgroups, including ATAF, ANAC3, NAP, ONAC022, NAM, NAC1, OsNAC7, ANAC001, TIP, ANAC011, NAC2, ONAC003, ANAC063, SENU5, and two unclassified KoNACs according to their homology with NAC proteins in *A. thaliana*; however, no KoNAC member from the TERN or OSNAC8 subfamily was identified (**Figure 1**). Among the 79 KoNAC proteins, the members belonging to the NAC and NAM subfamilies were the most abundant, with 10 members, whereas the ANAC3 subfamily contained the fewest KoNAC proteins, with only one member. The phylogenetic tree data indicated that KoNAC proteins showed some diversity, which was consistent with reports from *A. thaliana* (Ooka et al., 2003).

### Conserved motifs of protein KoNACs and gene structure of *KoNACs*

**Figure 2:**
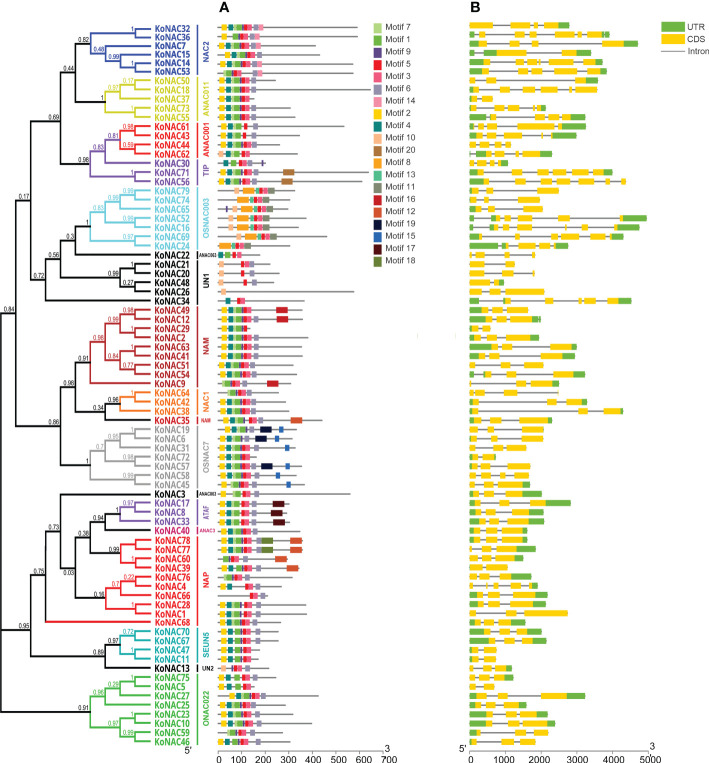
Conserved motifs of KoNACs and gene structures of *KoNACs* according to their phylogenetic relationships. The gene structures of *KoNACs* **(A)** were predicted with GSDS software. A green box represents the untranslated region (UTR), a yellow box represents an exon, and a black line represents an intron. The conserved motifs **(B)** were identified by the MEME web server. Each motif is indicated by a colored box, and the location of each motif can be estimated using the scale at the bottom.

To obtain more insights into the functional regions of KoNAC proteins, the conserved motifs were identified with the MEME program. A total of 20 conserved motifs were revealed, designated motifs 1–20 (**Supplementary Table S4**). These conserved motifs ranged in length from 8 to 50 amino acid residues. Furthermore, motifs 5, 3 and 1 were the conserved regions with the highest occurrence probability, and the frequencies of occurrence were 78, 66 and 62, respectively (**Figure 2A**). KoNAC26 contained only one type of motif, whereas KoNAC77 and KoNAC78 contained the greatest number of motifs (10 types). Generally, KoNAC proteins that were clustered in the same subgroups shared similar motif compositions, indicating that the same subgroups of genes showed similar functions.

To reveal the structural characteristics of the *KoNAC* genes, the intron–exon distribution pattern was analyzed using the program GSDS. The genomic structure of the *KoNACs* showed great variation, with the number of introns varying from 1 to 7 (**Figure 2B**). Among the 79 *KoNAC* genes, 9 (11.39%) had one exon, more than half (45, 56.96%) had two exons, and only 3 genes had more than 6 exons (*KoNAC16*, *KoNAC56* and *KoNAC36*, with 6, 6 and 7 exons, respectively).

### Chromosomal location, gene duplication, and synteny analysis of *KoNAC* genes

**Figure 3:**
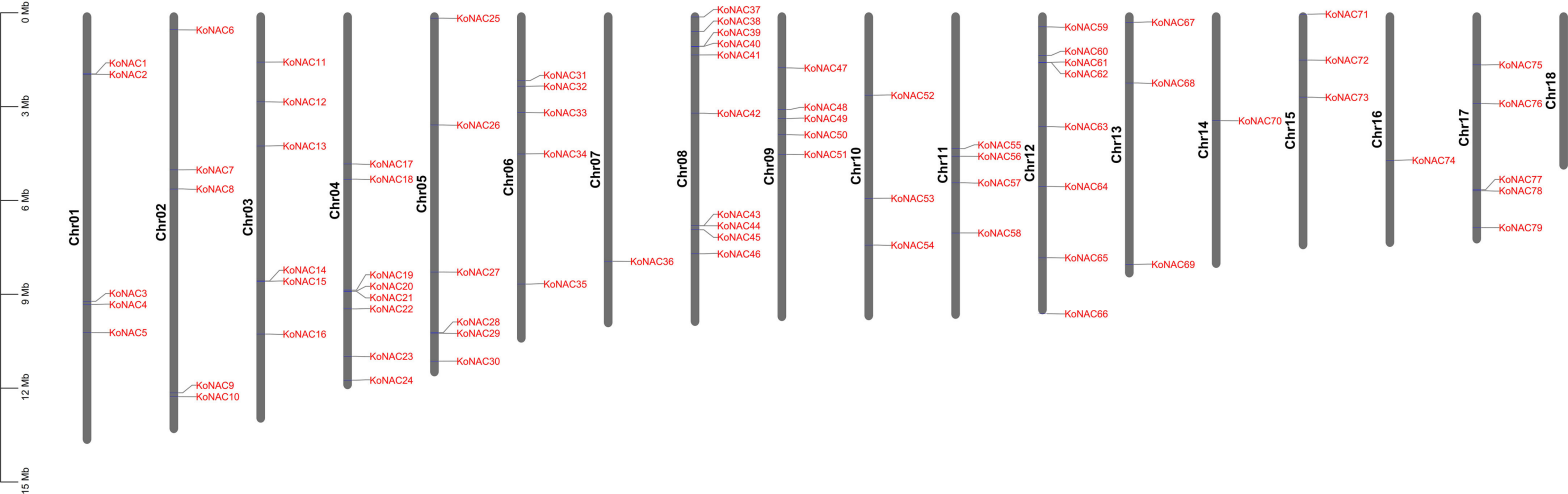
Distribution of 79 *KoNAC* genes on 18 chromosomes. Vertical bars represent the chromosomes of *Kandelia obovata*. The chromosome number is on the left of each chromosome. The scale on the left represents the length of the chromosome.

To clarify the distribution of *KoNAC* genes on the chromosomes of *K. obovata*, the program MapChart was used to map the chromosomal locations of all the identified *KoNAC* genes (**Figure 3**). The distribution of the 79 *KoNACs* throughout the 18 chromosomes was uneven, and there was no correlation between the number of genes on each chromosome and their length. Chromosome 8 contained the greatest number of *KoNAC* genes (10, 12.66%), and chromosomes 7, 14 and 16 harbored only one *KoNAC* gene each, whereas there were no *KoNAC* genes on chromosome 18.

**Figure 4:**
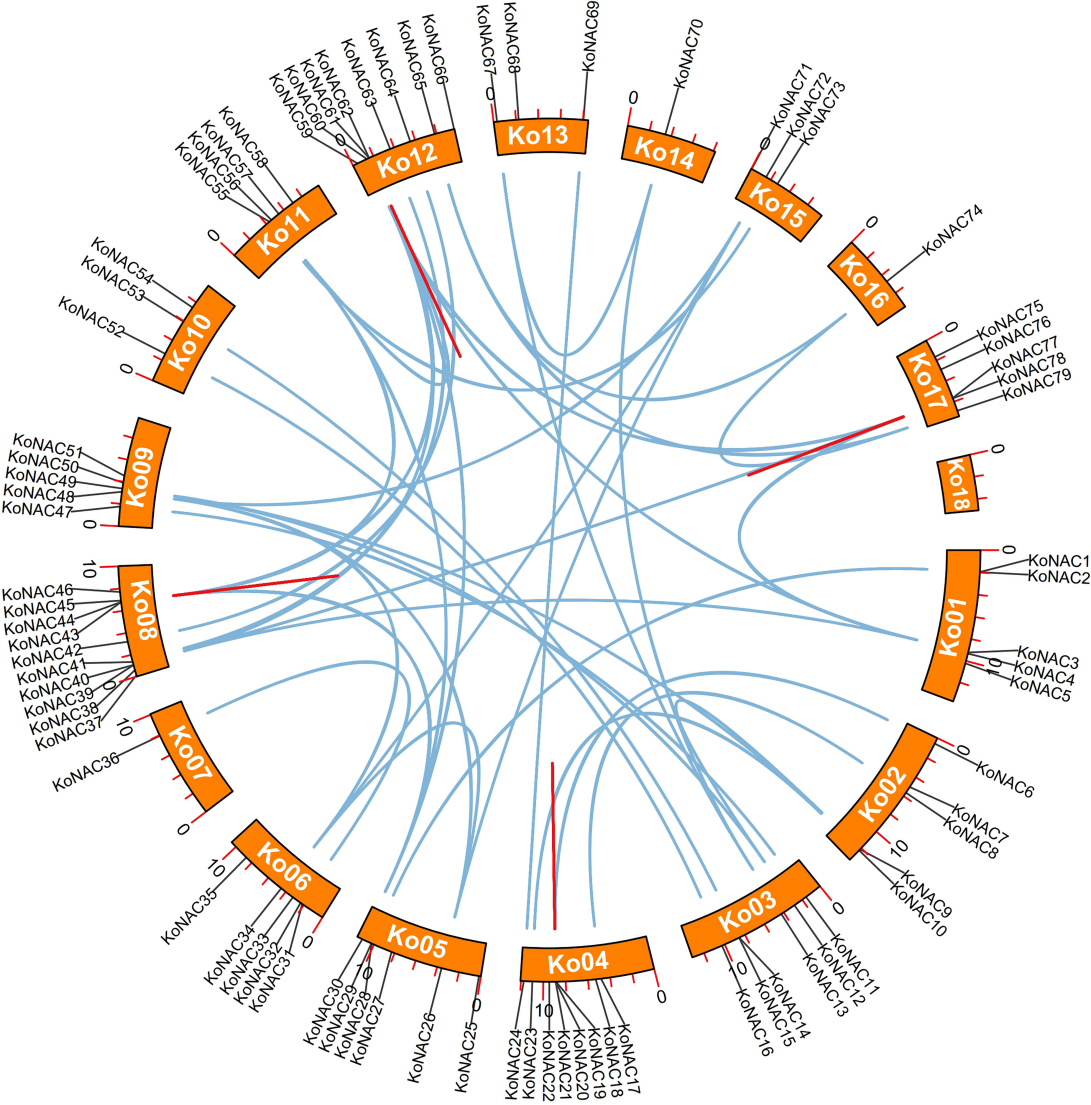
Schematic representations of the interchromosomal relationships of *KoNAC* genes. The blue lines indicate segmentally duplicated *KoNAC* gene pairs, and the red lines indicate tandemly duplicated *KoNAC* gene pairs.

To determine duplication events in the *KoNAC* genes, a synteny analysis was carried out using software MCScanX. Forty-nine pairs of segmentally duplicated genes were found among the 79 *KoNAC* members. Segmentally duplicated genes were the most common on chromosome 12, followed by chromosome 8, which contained 17 and 15 pairs, respectively (**Figure 4**, **Supplementary Table S5**). Four pairs of tandemly duplicated genes were identified in the *KoNAC* gene family, including KoNAC20/21, KoNAC43/44, KoNAC61/62, and KoNAC77/78, and the tandemly duplicated gene pairs were present on chromosomes 4, 8, 12, and 17, respectively (**Figure 4**, **Supplementary Table S5**). The results suggested that segmental duplication was mostly responsible for the expansion of the *KoNAC* genes.

To better understand the selective pressure on the *KoNAC* genes, the Ka/Ks ratios of the duplicated gene pairs were determined. All the segmentally and tandemly duplicated *KoNAC* gene pairs showed a Ka/Ks value < 1 (**Supplementary Table S6**), suggesting that strong purifying selection may have had a role in the evolution of the *KoNAC* gene family.

**Figure 5:**
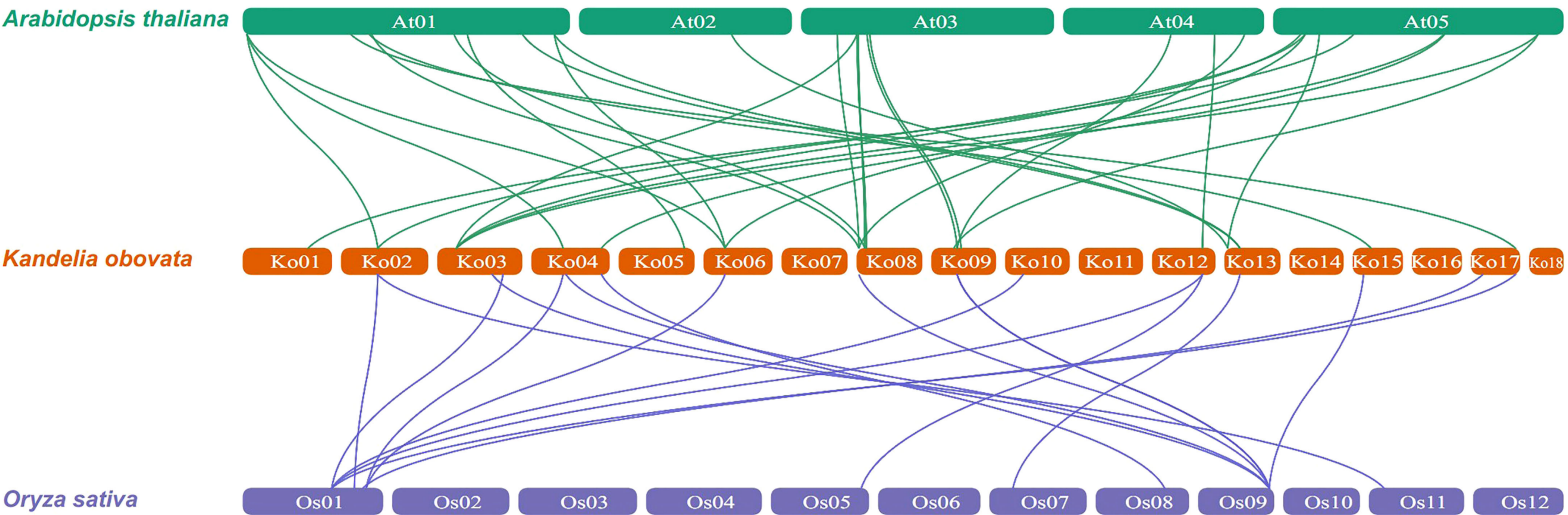
Synteny analysis of *NAC* genes between *Kandelia obovata* and two representative plant species (*Arabidopsis thaliana* and *Oryza sativa*). Green and blue lines represent syntenic *NAC* gene pairs of *K. obovata* and *A. thaliana* and *O. sativa*, respectively.

To further explore the evolutionary relationships of the *KoNAC* genes, comparative syntenic maps of the *A. thaliana* and *Oryza sativa* genes were constructed. The results indicated that 24 *KoNAC* genes showed collinearity relationships with 24 *NAC* genes in *A. thaliana* and 10 *NAC* genes in *O. sativa*. The number of orthologous gene pairs between *K. obovata* and *A. thaliana O. sativa* was 30, and that between *K. obovata* and *O. sativa* was 18 (**Figure 5**, **Supplementary Table S7**), suggesting that *NAC* genes underwent significant evolution and duplication after the divergence of dicotyledonous and monocotyledonous plants.

### *Cis*-elements in the promoter regions of *KoNAC* genes

**Figure 6:**
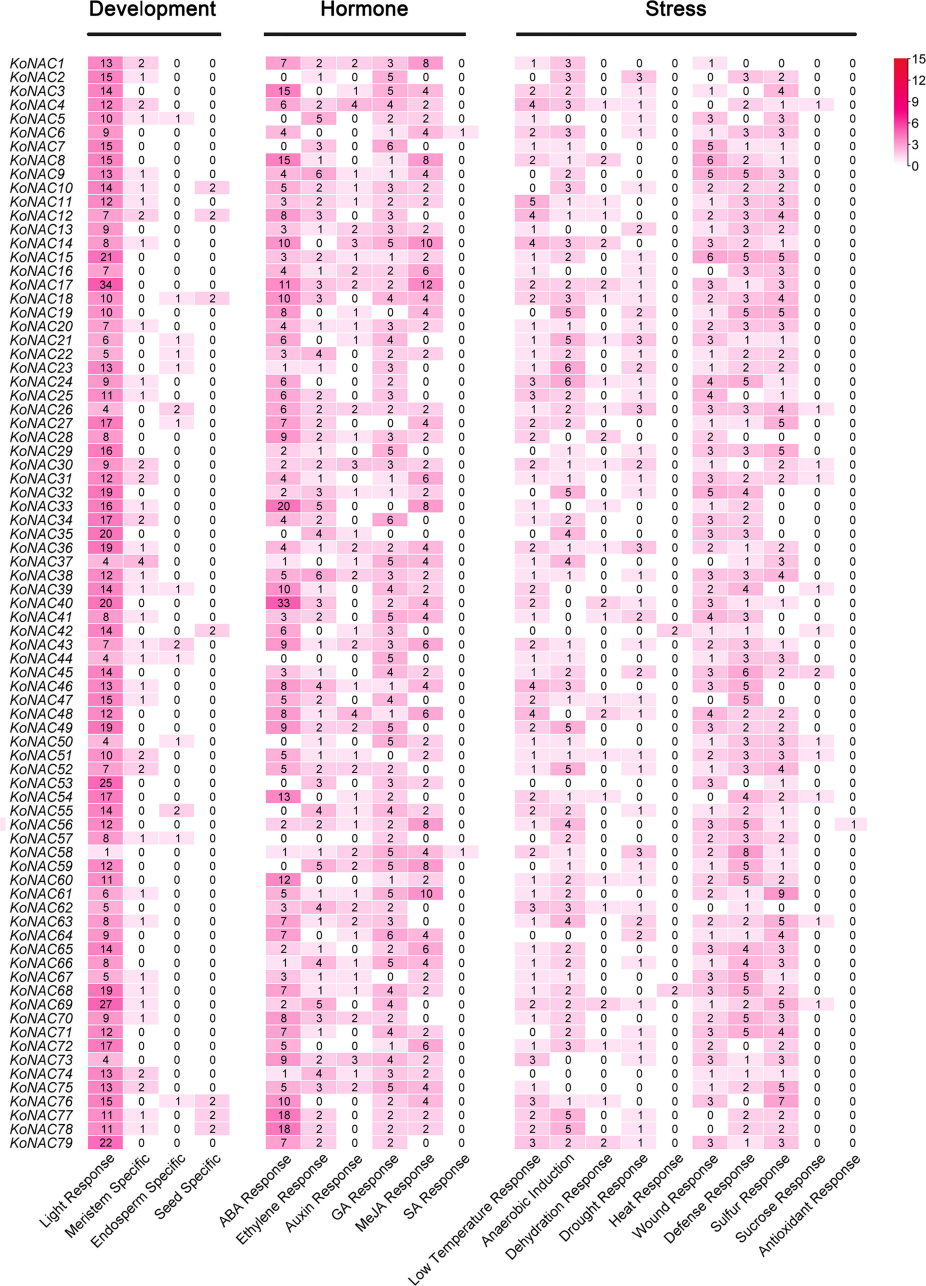
The number of each type of *cis*-acting element in the promoter region of each *KoNAC* gene. Annotation of the *cis*-elements: ABA, abscisic acid; GA, gibberellin; MeJA, methyl jasmonate; SA, salicylic acid.

To analyze the likely *cis*-elements of the *KoNAC* genes, the 2-kb upstream promoter region sequences of the *KoNACs* were obtained and used to search the PlantCARE database. A number of *cis*-elements related to developmental processes, phytohormone responses, and abiotic stress responses were identified (**Figure 6**, **Supplementary Figure S1**). The *cis*-elements related to plant development mainly included light-responsive elements and meristem-specific elements. The *cis*-elements involved in hormone responses included abscisic acid (ABA), gibberellin (GA), methyl jasmonate (MeJA), and ethylene response elements. Among the *cis*-elements related to stress responses, low-temperature response, anaerobic induction, and wound response elements were the most abundant motifs. The results suggested that *KoNAC*s might play an important role under the control of hormones during the development and stress responses of *K. obovata*.

### Confirmation of the subcellular localization of several KoNAC proteins

**Figure 7:**
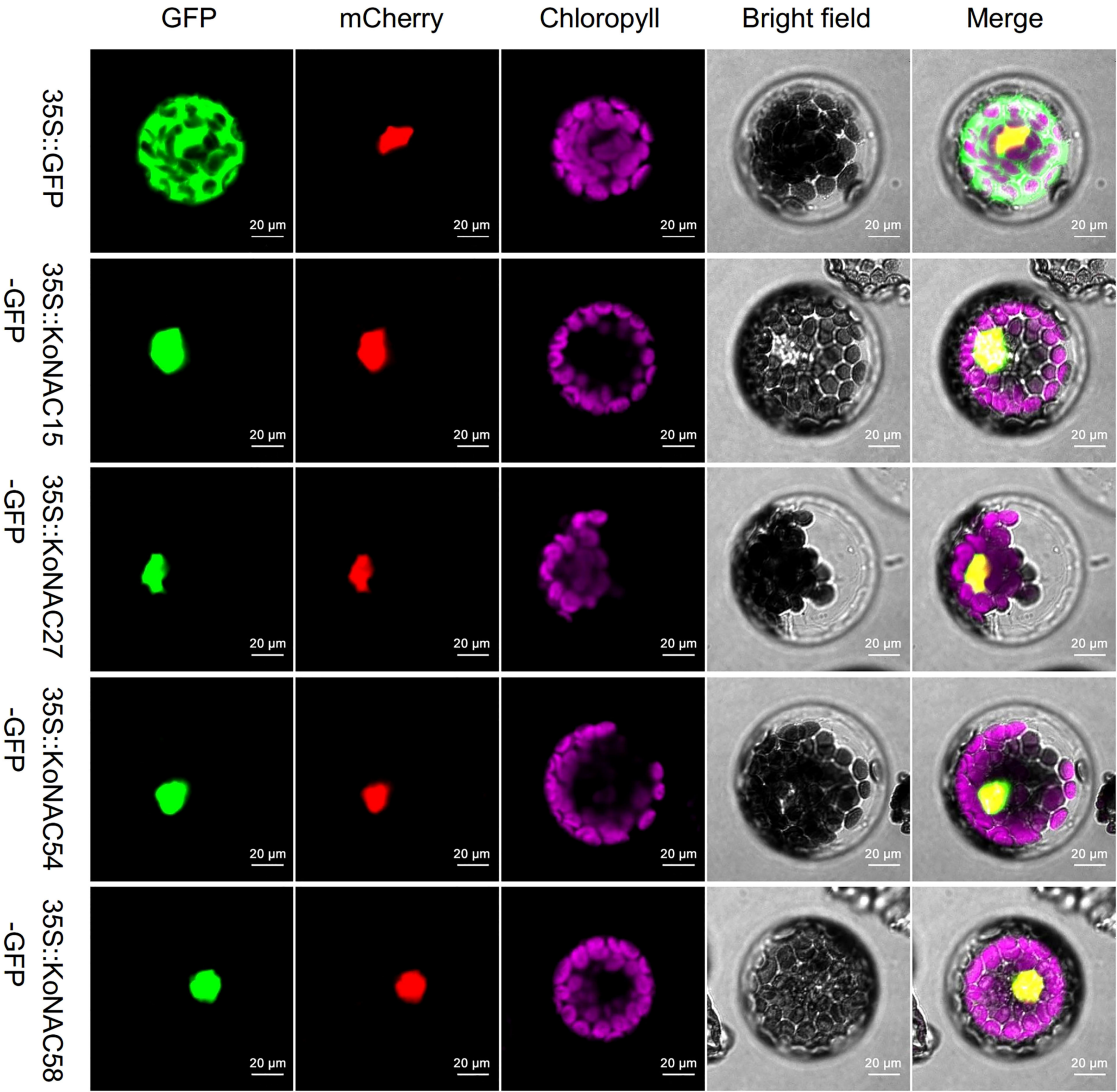
KoNAC-GFP fusion vectors were used to determine the subcellular localization of several KoNACs in *Arabidopsis thaliana* protoplast cells. The 35S::EGFP (green) construct used as a negative control was extensively observed in both the nucleus and the cytoplasm of *A. thaliana* protoplasts. A nuclear-localized 35S::mCherry protein (red) was applied to mark the nucleus. Chlorophyll autofluorescence (purple) demonstrates the location of chloroplasts. Scale bar = 0.02 mm.

To experimentally determine the subcellular localization of KoNACs, four members, KoNAC15, KoNAC27, KoNAC54, and KoNAC58, were randomly selected from different clades (NAC2, ONAC022, NAM, and OsNAC7, respectively) according to the previous phylogenetic tree, and their CDS were cloned into an expression vector containing the GFP tag driven by the CaMV35S promoter. Confocal microscopy analysis showed that the fluorescent signal of GFP was distributed in the plasma membrane, cytoplasm and nucleus of the protoplasts, while GFP-fused KoNAC15, KoNAC27, KoNAC54, and KoNAC58 localized only to the nucleus (**Figure 7**). Our results were consistent with the *in silico* subcellular localization prediction of KoNACs (**Supplementary Table S3**).

### Expression profiles of *KoNAC*s in different tissues

**Figure 8:**
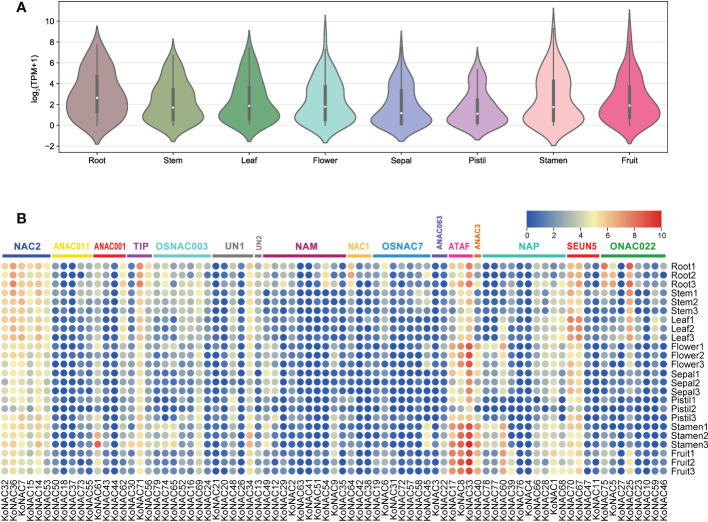
Expression of *KoNAC* genes in *Kandelia obovata*. **(A)** Bean plot of the expression levels of all *KoNACs* in different tissues. **(B)** The transcript levels of the *KoNAC* genes in 8 tissues of *K. obovata* were investigated based on publicly available transcriptomic data. The color scale shows increasing expression levels from blue to red.

The expression profiles of all 79 *KoNACs* in different tissues, such as the roots, stems, leaves, flowers, sepals, pistils, stamens, and fruit, were investigated based on *K. obovata* transcriptomic data that were publicly available. Generally, the average expression level of *KoNAC* genes was highest in roots, followed by stamens, and lowest in pistils (**Figure 8A**). Some *KoNAC* genes, such as *KoNAC25*, *KoNAC33*, *KoNAC36*, *KoNAC67*, *KoNAC70*, *KoNAC71*, and *KoNAC75*, showed high expression in roots; however, *KoNAC8*, *KoNAC14*, *KoNAC17*, *KoNAC33*, *KoNAC34*, *KoNAC40*, *KoNAC60*, and *KoNAC61* presented higher expression levels in stamens. Additionally, *KoNAC3*, *KoNAC4*, *KoNAC21*, *KoNAC26*, *KoNAC37*, *KoNAC41*, *KoNAC42*, *KoNAC44*, and *KoNAC76* were negligibly expressed in most of the tissues evaluated (**Figure 8B**). The variable expression patterns of *KoNACs* in different tissues may indicate that *KoNAC* genes play different roles during the tissue development process.

### Expression pattern of *KoNACs* in response to chilling stress

**Figure 9:**
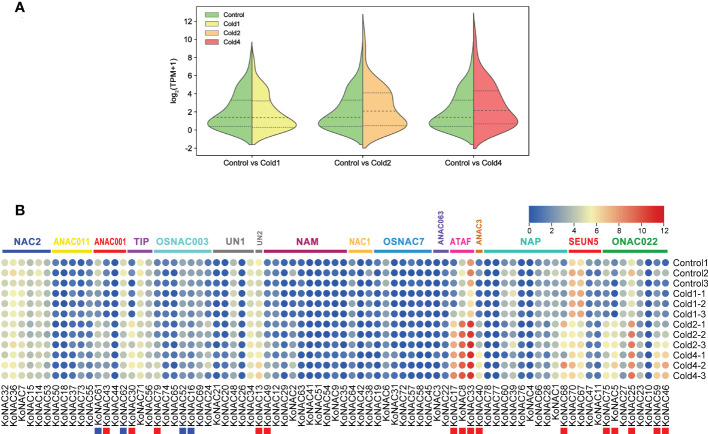
Expression of *KoNAC* genes in *Kandelia obovata*. **(A)** Bean plot of all *KoNACs* before and after 4°C chilling stress. **(B)** The transcript levels of *KoNAC* genes in response to cold stress were investigated based on publicly available transcriptomic data. Ten-month-old seedlings of *K. obovata* were treated, and the leaves were collected for transcriptome analysis. Cold1, the first time cold treatment, 2/5°C (light/day) for 4 d, then recovery at 22/27°C (light/day) for 15 d; Cold2, the second time cold treatment, continue 2/5°C (light/day) for 4 d, then recovery at 22/27°C (light/day) for 15 d; Cold3, the third time treatment, continue 2/5°C (light/day) for 4 d, then recovery at 22/27°C (light/day) for 15 d; Cold4, the fourth time treatment, continue 2/5°C (light/day) for 4 d. The red and blue squares indicate significantly enhanced and repressed expression under chilling stress, respectively (*p* < 0.05).

To evaluate the potential roles of *KoNACs* in response to cold stress, the expression pattern of *KoNACs* in the leaves of *K. obovata* was examined after being subjected to 5°C low temperature stress based on transcriptome data from NCBI database. In general, the average expression level of *KoNACs* did not change significantly compared with the control when subjected to the first time cold treatment; however, the second time and fourth time cold treatments significantly increased the expression of *KoNACs* (**Figure 9A**). The expression levels of most *KoNAC* genes, particularly *KoNAC5*, *KoNAC8*, *KoNAC13*, *KoNAC17*, *KoNAC25*, *KoNAC30*, *KoNAC33*, *KoNAC40*, *KoNAC43*, *KoNAC46*, *KoNAC49*, *KoNAC59*, *KoNAC68*, *KoNAC75*, and *KoNAC79*, were significantly upregulated more than twofold after chilling treatment. However, the expression levels of *KoNAC16*, *KoNAC52*, *KoNAC61*, and *KoNAC62* decreased with increasing chilling stress treatment time, particularly in the fourth time treatment, and the expression levels were significantly lower than those in the control (**Figure 9B**).

**Figure 10:**
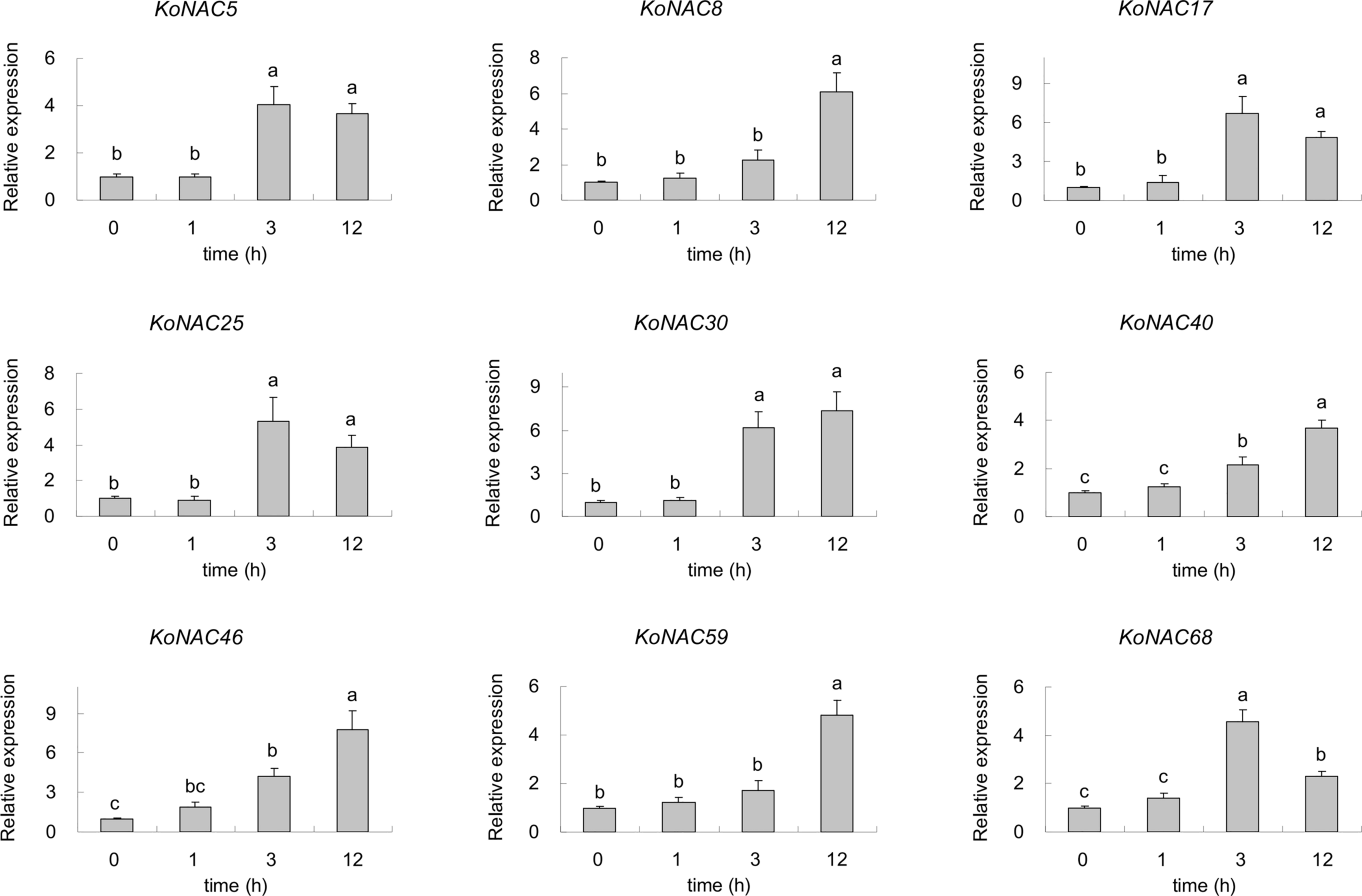
The expression profiles of nine potential *KoNACs* in response to chilling stress. The relative expression levels of nine *KoNACs* were measured in plants subjected to a temperature of 4°C for 0, 1, 3, and 12 h. The transcript levels of the selected genes were assessed *via* qRT**-**PCR and normalized to 18S rRNA levels. The error bars represent the standard errors. The values with the same letter are not significantly different according to Duncan’s multiple range test (*P* < 0.05; n =3).

To identify the potential *KoNACs* that are important to low-termpearture tolerance, nine upregulated *KoNACs* were chosen, and their relative expression levels were quantified by qRT-PCR. The expression of all the selected *KoNAC* genes was upregulated when subjected to 4°C stress for 3 h or 12 h compared to the control (**Figure 10**). Under low-temperature stress, the expression of *KoNAC5*, *KoNAC17*, *KoNAC25* and *KoNAC68* increased initially but then decreased, peaking at 3 h after 4°C treatment, and their expression levels were significantly higher than those in the control. However, the expression levels of *KoNAC8*, *KoNAC30*, *KoNAC40*, *KoNAC46* and *KoNAC59* continued to increase after 4°C treatment and peaked at 12 h.

### Significantly coexpressed gene pairs between *KoNACs* and mRNAs

Genome-wide gene expression profiling of *KoNACs* and mRNAs from the leaves of *K. obovata* treated with low temperature was conducted to find coexpressed gene pairs. The possible target mRNAs were determined using the Pearson correlation test for all abovementioned *KoNAC* genes whose expression significantly increased after chilling treatment. There was a correlation between 284 significantly expressed mRNAs and 13 *KoNACs* (|PCC| > 0.95; *P* < 0.001); all 381 coexpressed pairs were positively correlated except for KoNAC8-GWHTACBH001948 and KoNAC33-GWHTACBH018122, which were negatively correlated (**Supplementary Figure S2**; **Supplementary Table S8**). Overall, our results implied that the *KoNACs* might regulate the responses of these possible target genes to low-temperature stress mainly in a positive manner.

## Discussion

### Identification and evolutionary analysis of *KoNAC* genes

Many studies have reported that low temperatures are vital to the survival and distribution of mangroves (Krauss et al., 2008; Sippo et al., 2018; Fei et al., 2021a). NAC proteins are a class of TFs particular to plants that are vital for multiple plants during growth and development processes as well as in various biotic or abiotic defense responses (Nuruzzaman et al., 2013). An increasing number of *NAC* genes have been identified with the ongoing completion of genome sequencing in different species, such as *A. thaliana*, rice, tomato, barley, wheat, cotton, and potato (Yuan et al., 2019). Nevertheless, few NAC TFs engaged in the chilling stress response have been researched in *K. obovata* thus far. In the present study, the characteristics of *KoNAC* genes at the genome level were investigated, and their tissue expression profiles and responses to chilling stress were investigated. The goal of our study was to screen candidate *KoNAC* genes potentially engaged in the low-temperature response and provide a basis for the further elucidation of the functions and molecular mechanisms of *KoNAC* genes in response to chilling stress.

In the current study, seventy-nine *KoNAC* genes were identified in total in the whole genome of *K. obovata*, whose genome size was 180 Mb. It has been reported that the genomes of *A. thaliana*, *O. sativa* and *Fagopyrum tataricum*, with sizes of 125 Mb, 466 Mb and 489 Mb, contain 117, 151 and 80 *NAC* genes, respectively (Liu et al., 2019). The numbers of *NAC* genes are nearly the same in *K. obovata* and *F. tataricum*; however, the genome size of *F. tataricum* is more than 2.7 times that of *K. obovata*, so there is no correlation between the number of *NAC* genes in various plants and the size of their genome. Cannon et al. (2004) proposed that gene duplication can be considered the primary driving force of gene family expansion and evolution, where the main duplication patterns are segmental duplication and tandem duplication. It has been reported that tandem duplications play important roles in the expansion of the *NAC* family in *O. sativa* (Nuruzzaman et al., 2010), *Solanum tuberosum* (Singh et al., 2013) and *Eucalyptus grandis* (Hussey et al., 2015). Segmental duplications appear to be more dominant in the expansion of the *NAC* family, as observed in *Panicum miliaceum* (Shan et al., 2020), *Raphanus sativus* (Huang et al., 2022), and *Vigna radiate* (Tariq et al., 2022).

Our results indicated that 49 segmental duplications and 4 tandem duplications were detected in 79 *KoNAC* genes, which implied that segmental duplication was the main driving force for the expansion of the *KoNAC* gene family, similar to findings in *V. radiate* (Tariq et al., 2022). It was reported that *K. obovata* has undergone two polyploidization events: an ancient polyploidization event shared with most eudicots (γ-event) and a recent whole-genome duplication (WGD)/segmental duplication shared with other Rhizophoreae plants (Hu et al., 2020). However, larger segmental duplications occupy much of the *A. thaliana* genome, in which at least four large-scale duplication events occurred during the formative period in the diversification of angiosperms (100 to 200 million years ago) (Vision et al., 2000). This may be the reason why the *Arabidopsis* genome is smaller but has more NAC members than that of *K. obovata*.

The unrooted NJ tree based on NAC protein sequences of *K. obovata* and *A. thaliana* was constructed to explain the phylogenetic relationships, and all 79 *KoNAC* genes were divided into 16 subgroups according to their sequence homology with *A. thaliana* (Ooka et al., 2003). However, our results were not consistent with findings in other plant species, such as *Dactylis glomerata* (14 subgroups) (Yang et al., 2021), *Fagopyrum tataricum* (15 subgroups) (Liu et al., 2019), *Chenopodium quinoa* (14 subgroups) (Li et al., 2019), and *Theobroma cacao* (12 subgroups) (Shen et al., 2020), suggesting that NAC proteins exhibit diversity in different species. These differences may be associated with the fact that the above four species are all terrestrial plants, while *K. obovata* is a mangrove plant. Thus, more NAC transcription factors may have evolved over a long period of time to allow the mangrove species to adapt to harsher habitats characterized by high salinity, submergence, and hypoxia.

The number of introns in the *KoNAC* genes varied from 1 to 7, similar to numbers reported in many plant species (Liu et al., 2019; Shan et al., 2020; Song et al., 2022). In general, the deletion or insertion of introns can result in a loss of gene function. Interestingly, the intron numbers of *KoNAC* genes were significantly negatively correlated with the percent changes in their expression levels after the first cold treatment (**Supplementary Figure S3**). Jeffares et al. (2008) showed that genes with rapidly changing expression levels in response to stress present significantly lower intron densities in *A. thaliana*; perhaps the splicing of two or more introns requires more time than transcription and becomes rate limiting. Our research is consistent with the results of Jeffares, indicating that more attention should be given to the *KoNACs* with fewer introns if the research goal is to focus on genes that are immediately responsive to environmental stress.

### Expression profiles and functional prediction of *KoNAC* genes

As one of the largest families of TFs, NACs act as positive or negative regulators that regulate the responses of plant species such as *A. thaliana* and rice to biotic and abiotic stresses (Ooka et al., 2003; Nuruzzaman et al., 2013). In the present study, expression analyses revealed that *KoNAC7*, *KoNAC14*, *KoNAC33*, *KoNAC67*, and *KoNAC71* showed constitutively high expression levels in root, stem, leaf, pistil, stamen, flower, sepal, and fruit, implying that they may play important roles in different tissues of the plant; this result is similar to the finding that *FtNAC14* from *F. tataricum* was most abundantly expressed in both stem and leaf, *FtNAC43*, *FtNAC46* and *FtNAC58* were highly expressed in the root; however, *FtNAC70* showed the highest expression level in the flower (Liu et al., 2019). Moreover, *KoNAC45* and *KoNAC54* specifically displayed high expression in reproductive or vegetative organs, showing high expression in stamens and roots, respectively; a similar result was found in *Picea wilsonii*, in which *PwNAC30* exhibits tissue-specific expression, mainly in roots, leaves, pistils, and pods but not in stamens (Liang et al., 2020). Taken together, our results suggested the differential and specific roles of *KoNAC* family members during *K. obovata* growth and development.

Many studies have been conducted to clarify the function of NAC TFs in reactions to various environmental stresses, such as salinity, drought, heat and flooding (Fang et al., 2015; Yuan et al., 2020; Song et al., 2022). In the present study, the average expression level of the *KoNACs* was highest in roots, and a large number of *KoNAC* genes showed high levels of expression, such as *KoNAC25*, *KoNAC33*, *KoNAC36*, *KoNAC64*, *KoNAC67*, *KoNAC70*, *KoNAC71*, and *KoNAC75* (**Figure 8B**). The plant root is an organ that directly contacts the soil and is the first line of defense for maintaining plant productivity under soil abiotic stress (Suralta et al., 2018). Liu et al. (2016) reported that the overexpression of *A. thaliana ATAF1* (AT1g01720) in rice increased its root length, shoot height and tolerance to salt. *KoNAC33* is an ortholog of *ATAF1* (**Figure 1**), and *K. obovata* survives in harsh environments characterized by conditions such as submergence, hypoxia, and salinity (Fei et al., 2021b; Nizam et al., 2022); thus, the high expression levels of *KoNACs* may alleviate the effects of environmental stresses. Further studies on *KoNACs* are needed to determine their functions and regulatory mechanisms.

RNA-seq results indicated that the expression levels of some *KoNACs* in the leaves of *K. obovata* changed significantly under low-temperature stress. Among these *KoNACs*, fifteen members were found to be upregulated more than twofold, but only 4 were downregulated. Similar outcomes were discovered in *Pyrus pyrifolia* (Ahmad et al., 2018), *P. bretschneideri* (Gong et al., 2019), and *Prunus mume* (Zhuo et al., 2018), meaning that *NACs* might act as positive or negative regulators. Overexpression of *LlNAC2* (from *Lilium lancifolium*) and *CaNAC035* (from *Capsicum annuum*) in *A. thaliana* enhances tolerance to chilling stress (Yong et al., 2019; Zhang et al., 2020), and the same function was also reported in a study of the *SlNAC1* gene from *Suaeda liaotungensis* (Li et al., 2014). Previous studies have offered convincing support for the phylogenetic analysis-based prediction of the functions of several TF gene family members (Yuan et al., 2020). Both *LlNAC2* and *CaNAC035* belong to the ATAF subfamily, and these genes are homologous to *KoNAC8*, *KoNAC17*, and *KoNAC33*; moreover, *SlNAC1* is clustered into the TIP subgroup and is homologous to *KoNAC30*, indicating that *KoNAC8*, *KoNAC17*, *KoNAC30*, and *KoNAC33* may play an important role in the response to chilling stress in *K. obovata*.

In PCC analysis, a total of 10 WRKY, 4 ERF, 1 GATA, 2 MYB-related proteins and 2 zinc finger TFs were found to be positively correlated with the expression of NACs, suggesting that a variety of TFs were involved in the adaptation process when *K. obovata* was subjected to low-temperature stress. Recently, Fei et al. (2022) reported that *KoWRKY40* was highly induced in leaves and roots under 5°C cold stress, ectopic overexpression of the *KoWRKY40* gene in *A. thaliana* significantly enhanced the low-temperature tolerance of transgenic plants and increased the expression of antioxidant enzyme and *COR* genes. *KoWRKY40* is highly homologous to GWHTACBH004052 in this study, which is significantly and positively correlated with the expression of *KoNAC25*, *KoNAC8* and *KoNAC33*. In addition, Sun et al. (2019) showed that the transcription factor VaERF092 regulates the expression of *VaWRKY33* from *Vitis amurensis* by binding to the promoter GCC-box of *VaWRKY33*, leading to the enhancement of transgenic grape calli to cold stress. Therefore, further studies conducted to elucidate the precise regulatory mechanisms of TF genes in response to chilling stress should consider the interactions between TFs above.

## Conclusion

A total of 79 *KoNAC* genes were systematically identified and characterized in *K. obovata*, and their expression patterns in eight tissues and their expression profiles under low temperature were analyzed. These TFs could be categorized into 16 subgroups based on the phylogenetic tree constructed with NAC proteins of *A. thaliana*. Gene chromosomal location and synteny analysis showed that segmental duplication was the main driving force of the expansion of *KoNAC* genes. RNA-seq data indicated tissue-specific expression of some *NACs*, and 15 members were upregulated significantly in response to cold stress, but only 4 were downregulated. The *KoNACs* with significantly changed expression levels may be candidate genes for the genetic engineering of *K. obovata* with enhanced stress tolerance. Our research provides systematic information about *KoNAC* genes and will facilitate more functional studies of prospective *KoNAC* members in response to abiotic stress.

## Data availability statement

Publicly available datasets were analyzed in this study. This data can be found here: https://bigd.big.ac.cn/gsa/browse/CRA002395.

## Author contributions

ZD and JL designed the research study. ZD, SY, DY, YZ, and ZC performed the experiments. ZD, YT, and WS analyzed the data. ZD and JL wrote the manuscript. All authors contributed to editorial changes in the manuscript. All authors read and approved the final manuscript.

## Funding

This study was supported by the Public Welfare Project of Science Technology Department of Zhejiang Province (LGN21C160007), the Science and Technology Project of Department of Natural Resources of Zhejiang Province (2021-43), Taizhou Science and Technology Project (22nya05), and the Project of Yuhuan Municipal Bureau of Natural Resources and Planning (BY-YHGQCG-2021-04).

## Conflict of interest

Author ZC was employed by Taizhou Circular Economy Development Co., Ltd.

The remaining authors declare that the research was conducted in the absence of any commercial or financial relationships that could be construed as a potential conflict of interest.

## Publisher’s note

All claims expressed in this article are solely those of the authors and do not necessarily represent those of their affiliated organizations, or those of the publisher, the editors and the reviewers. Any product that may be evaluated in this article, or claim that may be made by its manufacturer, is not guaranteed or endorsed by the publisher.

## Supplementary material

Supplementary Figure 1
*cis*-acting elements in each *KoNAC*. The distribution of 20 *cis*-acting elements in the 2000 bp upstream promoter region is shown. The different types of *cis*-acting elements are represented by different colors, as shown on the right.

Supplementary Figure 2 Coexpression network of *KoNACs* with other genes in *Kandelia obovata* under chilling stress. The coexpression network was established between 13 *KoNACs* and 284 significantly expressed mRNAs whose absolute Pearson correlation coefficient values were equal to or greater than 0.95. Within this coexpression network, 379 and 2 pairs were positively and negatively correlated, respectively. The yellow circles represent *KoNAC* genes, while the blue rectangles represent the coexpressed genes. The solid gray lines represent positive correlations, while the dashed green lines represent negative correlations.

Supplementary Figure 3 The percent change in the expression levels of *KoNACs* under the first cold treatment is negatively correlated with the number of introns. The percent change in the expression levels of *KoNACs* under the first cold treatment was calculated with the formula (E_1_-E_0_)*100%/E_0_. E_1_ and E_0_ indicate the expression levels of *KoNACs* under the first cold treatment and in the control, respectively.

## References

[B1] Ahmad M. Yan X. Li J. Yang Q. Jamil W. Teng Y. . (2018). Genome wide identification and predicted functional analyses of NAC transcription factors in Asian pears. BMC Plant Biol. 18, 214. doi: 10.1186/s12870-018-1427-x 30285614 PMC6169067

[B2] Bardou R. Parker J. D. Feller I. C. Cavanaugh K. C. (2020). Variability in the fundamental versus realized niches of north American mangroves. J. Biogeography. 48, 160–175. doi: 10.1111/jbi.13990

[B3] Cannon S. B. Mitra A. Baumgarten A. Young N. D. May G. (2004). The roles of segmental and tandem gene duplication in the evolution of large gene families in *Arabidopsis thaliana* . BMC Plant Biol. 4, 10. doi: 10.1186/1471-2229-4-10 15171794 PMC446195

[B4] Chen C. Chen H. Zhang Y. Thomas H. R. Frank M. H. He Y. . (2020). TBtools: an integrative toolkit developed for interactive analyses of big biological data. Mol. Plant 13, 1194–1202. doi: 10.1016/j.molp.2020.06.009 32585190

[B5] Chen L. Wang W. Li Q. Q. Zhang Y. Yang S. Osland M. J. . (2017). Mangrove species' responses to winter air temperature extremes in China. Ecosphere. 8, e01865. doi: 10.1002/ecs2.1865

[B6] Diao P. Chen C. Zhang Y. Meng Q. Lv W. Ma N. (2020). The role of NAC transcription factor in plant cold response. Plant Signaling Behavior. 15, 1785668. doi: 10.1080/15592324.2020.1785668 32662739 PMC8550289

[B7] Dong Y. Tang M. Huang Z. Song J. Xu J. Ahammed G. J. . (2022). The miR164a-NAM3 module confers cold tolerance by inducing ethylene production in tomato. Plant J. 111, 440–456. doi: 10.1111/tpj.15807 35569132

[B8] Du Z. Li J. (2019). Expression, purification and molecular characterization of a novel transcription factor KcCBF3 from *Kandelia candel* . Protein Expression Purification. 153, 26–34. doi: 10.1016/j.pep.2018.08.006 30118861

[B9] Du Z. You S. Zhao X. Xiong L. Li J. (2022). Genome-wide identification of *WRKY* genes and their responses to chilling stress in *Kandelia obovata* . Front. Genet. 13, 875316. doi: 10.3389/fgene.2022.875316 35432463 PMC9008847

[B10] Fang Y. Liao K. Du H. Xu Y. Song H. Li X. . (2015). A stress-responsive NAC transcription factor SNAC3 confers heat and drought tolerance through modulation of reactive oxygen species in rice. J. Exp. Botany. 66, 6803–6817. doi: 10.1093/jxb/erv386 26261267 PMC4623689

[B11] Fei J. Wang Y.-S. Cheng H. Sun F.-L. Sun C.-C. (2021a). Comparative physiological and proteomic analyses of mangrove plant *Kandelia obovata* under cold stress. Ecotoxicology. 30, 1826–1840. doi: 10.1007/s10646-021-02483-6 34618290

[B12] Fei J. Wang Y.-S. Cheng H. Su Y.-B. Zhong Y. Zheng L. (2021b). Cloning and characterization of *KoOsmotin* from mangrove plant *Kandelia obovata* under cold stress. BMC Plant Biol. 21, 10. doi: 10.1186/s12870-020-02746-0 33407136 PMC7789355

[B13] Fei J. Wang Y.-S. Cheng H. Su Y.-B. Zhong Y.-J. Zheng L. (2022). The *Kandelia obovata* transcription factor *KoWRKY40* enhances cold tolerance in transgenic *Arabidopsis* . BMC Plant Biol. 22, 274. doi: 10.1186/s12870-022-03661-2 35659253 PMC9166612

[B14] Fei J. Wang Y.-S. Jiang Z.-Y. Cheng H. Zhang J.-D. (2015). Identification of cold tolerance genes from leaves of mangrove plant *Kandelia obovata* by suppression subtractive hybridization. Ecotoxicology 24, 1686–1696. doi: 10.1007/s10646-015-1486-9 26002218

[B15] Gong X. Zhao L. Song X. Lin Z. Gu B. Yan J. . (2019). Genome-wide analyses and expression patterns under abiotic stress of NAC transcription factors in white pear (*Pyrus bretschneideri*). BMC Plant Biol. 19, 161. doi: 10.1186/s12870-019-1760-8 31023218 PMC6485137

[B16] Guo W. Wu H. Zhang Z. Yang C. Hu L. Shi X. . (2017). Comparative analysis of transcriptomes in rhizophoraceae provides insights into the origin and adaptive evolution of mangrove plants in intertidal environments. Front. Plant Sci. 8, 795. doi: 10.3389/fpls.2017.00795 28559911 PMC5432612

[B17] Huang Y. Cui L. Chen W. Liu Z. Yuan W. Zhu F. . (2022). Comprehensive analysis of NAC transcription factors and their expressions during taproot coloration in radish (*Raphanus sativus* L.). Scientia Horticulturae. 299, 111047. doi: 10.1016/j.scienta.2022.111047

[B18] Hussey S. G. Saïdi M. N. Hefer C. A. Myburg A. A. Grima-Pettenati J. (2015). Structural, evolutionary and functional analysis of the NAC domain protein family in *Eucalyptus* . New Phytologist. 206, 1337–1350. doi: 10.1111/nph.13139 25385212

[B19] Hu M.-J. Sun W.-H. Tsai W.-C. Xiang S. Lai X.-K. Chen D.-Q. . (2020). Chromosome-scale assembly of the *Kandelia obovata* genome. Horticulture Res. 7, 75. doi: 10.1038/s41438-020-0300-x PMC7195387 32377365

[B20] Jeffares D. C. Penkett C. J. Bähler J. (2008). Rapidly regulated genes are intron poor. Trends Genet. 24, 375–378. doi: 10.1016/j.tig.2008.05.006 18586348

[B21] Jiang J. Hou R. Yang N. Li L. Deng J. Qin G. . (2021). Physiological and TMT-labeled proteomic analyses reveal important roles of sugar and secondary metabolism in *Citrus junos* under cold stress. J. Proteomics. 237, 104145. doi: 10.1016/j.jprot.2021.104145 33581353

[B22] Jin C. Li K.-Q. Xu X.-Y. Zhang H.-P. Chen H.-X. Chen Y.-H. . (2017). A novel NAC transcription factor, *PbeNAC1*, of *Pyrus betulifolia* confers cold and drought tolerance *via* interacting with *PbeDREBs* and activating the expression of stress-responsive genes. Front. Plant Science. 8, 1049. doi: 10.3389/fpls.2017.01049 PMC5491619 28713394

[B23] Krauss K. W. Lovelock C. E. McKee K. L. López-Hoffman L. Ewe S. M. Sousa W. P. (2008). Environmental drivers in mangrove establishment and early development: A review. Aquat. Botany. 89, 105–127. doi: 10.1016/j.aquabot.2007.12.014

[B24] Liang K.-h. Wang A.-b. Yuan Y.-h. Miao Y.-h. Zhang L.-y. (2020). *Picea wilsonii* NAC transcription factor PwNAC30 negatively regulates abiotic stress tolerance in transgenic *Arabidopsis* . Plant Mol. Biol. Reporter. 38, 554–571. doi: 10.1007/s11105-020-01216-z

[B25] Li F. Guo X. Liu J. Zhou F. Liu W. Wu J. . (2019). Genome-wide identification, characterization, and expression analysis of the NAC transcription factor in *Chenopodium quinoa* . Genes. 10, 500. doi: 10.3390/genes10070500 31262002 PMC6678211

[B26] Liu M. Ma Z. Sun W. Huang L. Wu Q. Tang Z. . (2019). Genome-wide analysis of the NAC transcription factor family in tartary buckwheat (*Fagopyrum tataricum*). BMC Genomics 20, 113. doi: 10.1186/s12864-019-5500-0 30727951 PMC6366116

[B27] Liu Y. Sun J. Wu Y. (2016). *Arabidopsis ATAF1* enhances the tolerance to salt stress and ABA in transgenic rice. J. Plant Res. 129, 955–962. doi: 10.1007/s10265-016-0833-0 27216423

[B28] Livak K. J. Schmittgen T. D. (2001). Analysis of relative gene expression data using real-time quantitative PCR and the 2^–ΔΔCT^ method. Methods. 25, 402–408. doi: 10.1006/meth.2001.1262 11846609

[B29] Li X.-l. Yang X. Hu Y.-x. Yu X.-d. Li Q.-l. (2014). A novel NAC transcription factor from *Suaeda liaotungensis* k. enhanced transgenic *Arabidopsis* drought, salt, and cold stress tolerance. Plant Cell Rep. 33, 767–778. doi: 10.1007/s00299-014-1602-y 24682461

[B30] Lu W.-X. Zhang B.-H. Zhang Y.-Y. Yang S.-C. (2022). Differentiation of cold tolerance in an artificial population of a mangrove species, *Kandelia obovata*, is associated with geographic origins. Front. Plant Science. 12, 695746. doi: 10.3389/fpls.2021.695746 PMC8851163 35185942

[B31] Manna M. Thakur T. Chirom O. Mandlik R. Deshmukh R. Salvi P. (2021). Transcription factors as key molecular target to strengthen the drought stress tolerance in plants. Physiologia Plantarum. 172, 847–868. doi: 10.1111/ppl.13268 33180329

[B32] Nakashima K. Takasaki H. Mizoi J. Shinozaki K. Yamaguchi-Shinozaki K. (2012). NAC transcription factors in plant abiotic stress responses. Biochim. Biophys. Acta 1819, 97–103. doi: 10.1016/j.bbagrm.2011.10.005 22037288

[B33] Nakashima K. Tran L.-S.P. Van Nguyen D. Fujita M. Maruyama K. Todaka D. (2007). Functional analysis of a NAC-type transcription factor OsNAC6 involved in abiotic and biotic stress-responsive gene expression in rice. Plant J. 51, 617–630. doi: 10.1111/j.1365-313X.2007.03168.x 17587305

[B34] Nizam A. Meera S. P. Kumar A. (2022). Genetic and molecular mechanisms underlying mangrove adaptations to intertidal environments. iScience. 25, 103547. doi: 10.1016/j.isci.2021.103547 34988398 PMC8693430

[B35] Nuruzzaman M. Manimekalai R. Sharoni A. M. Satoh K. Kondoh H. Ooka H. . (2010). Genome-wide analysis of NAC transcription factor family in rice. Gene. 465, 30–44. doi: 10.1016/j.gene.2010.06.008 20600702

[B36] Nuruzzaman M. Sharoni A. M. Kikuchi S. (2013). Roles of NAC transcription factors in the regulation of biotic and abiotic stress responses in plants. Front. Microbiol. 4, 248. doi: 10.3389/fmicb.2013.00248 24058359 PMC3759801

[B37] Olsen A. N. Ernst H. A. Leggio L. L. Skriver K. (2005). NAC transcription factors: Structurally distinct, functionally diverse. Trends Plant Science. 10, 79–87. doi: 10.1016/j.tplants.2004.12.010 15708345

[B38] Ooka H. Satoh K. Doi K. Nagata T. Otomo Y. Murakami K. . (2003). ). comprehensive analysis of NAC family genes in *Oryza sativa* and *Arabidopsis thaliana* . DNA Res. 10, 239–247. doi: 10.1093/dnares/10.6.239 15029955

[B39] Osland M. J. Enwright N. Day R. H. Doyle T. W. (2013). Winter climate change and coastal wetland foundation species: Salt marshes vs. mangrove forests in the southeastern united states. Global Change Biol. 19, 1482–1494. doi: 10.1111/gcb.12126 23504931

[B40] Peng Y.-L. Wang Y.-S. Fei J. Sun C.-C. (2020). Isolation and expression analysis of two novel c-repeat binding factor (*CBF*) genes involved in plant growth and abiotic stress response in mangrove Kandelia obovata. Ecotoxicology 29, 718–725. doi: 10.1007/s10646-020-02219-y 32394360

[B41] Peng Y.-L. Wang Y.-S. Fei J. Sun C.-C. Cheng H. (2015). Ecophysiological differences between three mangrove seedlings (*Kandelia obovata*, *Aegiceras corniculatum*, and *Avicennia marina*) exposed to chilling stress. Ecotoxicology. 24, 1722–1732. doi: 10.1007/s10646-015-1488-7 26002219

[B42] Shan Z. Jiang Y. Li H. Guo J. Dong M. Zhang J. . (2020). Genome-wide analysis of the NAC transcription factor family in broomcorn millet (*Panicum miliaceum* L.) and expression analysis under drought stress. BMC Genomics 21, 96. doi: 10.1186/s12864-020-6479-2 32000662 PMC6993341

[B43] Shao H. Wang H. Tang X. (2015). NAC transcription factors in plant multiple abiotic stress responses: progress and prospects. Front. Plant Sci. 6, 902. doi: 10.3389/fpls.2015.00902 26579152 PMC4625045

[B44] Shen Z.-J. Qin Y.-Y. Luo M.-R. Li Z. Ma D.-N. Wang W.-H. . (2021). Proteome analysis reveals a systematic response of cold-acclimated seedlings of an exotic mangrove plant *Sonneratia apetala* to chilling stress. J. Proteomics. 248, 104349. doi: 10.1016/j.jprot.2021.104349 34411764

[B45] Shen S. Zhang Q. Shi Y. Sun Z. Zhang Q. Hou S. . (2020). Genome-wide analysis of the NAC domain transcription factor gene family in *Theobroma cacao* . Genes. 11, 35. doi: 10.3390/genes11010035 PMC7017368 31905649

[B46] Sheue C.-R. Liu H.-Y. Yong J. W. H. (2003). *Kandelia obovata* (Rhizophoraceae), a new mangrove species from Eastern Asia. Taxon. 52, 287–294. doi: 10.2307/3647398

[B47] Singh S. Koyama H. Bhati K. K. Alok A. (2021). The biotechnological importance of the plant-specific NAC transcription factor family in crop improvement. J. Plant Res. 134, 475–495. doi: 10.1007/s10265-021-01270-y 33616799 PMC8106581

[B48] Singh A. K. Sharma V. Pal A. K. Acharya V. Ahuja P. S. (2013). Genome-wide organization and expression profiling of the NAC transcription factor family in potato (*Solanum tuberosum* L.). DNA Res. 20, 403–423. doi: 10.1093/dnares/dst019 23649897 PMC3738166

[B49] Sippo J. Z. Lovelock C. E. Santos I. R. Sanders C. J. Maher D. T. (2018). Mangrove mortality in a changing climate: An overview. Estuarine Coast. Shelf Sci. 215, 241–249. doi: 10.1016/j.ecss.2018.10.011

[B50] Song Y. Cui H. Shi Y. Xue J. Ji C. Zhang C. . (2020). Genome-wide identification and functional characterization of the *Camelina sativa WRKY* gene family in response to abiotic stress. BMC Genomics 21, 786. doi: 10.1186/s12864-020-07189-3 33176698 PMC7659147

[B51] Song H. Liu Y. Dong G. Zhang M. Wang Y. Xin J. . (2022). Genome-wide characterization and comprehensive analysis of NAC transcription factor family in *Nelumbo nucifera* . Front. Genet. 13, 901838. doi: 10.3389/fgene.2022.901838 35754820 PMC9214227

[B52] Sun X. Zhang L. Wong D. C. J. Wang Y. Zhu Z. Xu G. . (2019). The ethylene response factor VaERF092 from amur grape regulates the transcription factor VaWRKY33, improving cold tolerance. Plant J 99, 988–1002. doi: 10.1111/tpj.14378 31063661

[B53] Suralta R. R. Kano-Nakata M. Niones J. M. Inukai Y. Kameoka E. Tran T. T. . (2018). Root plasticity for maintenance of productivity under abiotic stressed soil environments in rice: Progress and prospects. Field Crops Res. 220, 57–66. doi: 10.1016/j.fcr.2016.06.023

[B54] Su W. Ye C. Zhang Y. Hao S. Li Q. Q. (2019). Identification of putative key genes for coastal environments and cold adaptation in mangrove *Kandelia obovata* through transcriptome analysis. Sci. Total Environment. 681, 191–201. doi: 10.1016/j.scitotenv.2019.05.127 31103657

[B55] Tariq R. Hussain A. Tariq A. Khalid M. H. B. Khan I. Basim H. . (2022). Genome-wide analyses of the mung bean NAC gene family reveals orthologs, co-expression networking and expression profiling under abiotic and biotic stresses. BMC Plant Biol. 22, 343. doi: 10.1186/s12870-022-03716-4 35836131 PMC9284730

[B56] Trégarot E. Caillaud A. Cornet C. C. Taureau F. Catry T. Cragg S. M. . (2021). Mangrove ecological services at the forefront of coastal change in the French overseas territories. Sci. Total Environment. 763, 143004. doi: 10.1016/j.scitotenv.2020.143004 33158516

[B57] Vision T. J. Brown D. G. Tanksley S. D. (2000). The origins of genomic duplications in *Arabidopsis* . Science. 290, 2114–2117. doi: 10.1126/science.290.5499.2114 11118139

[B58] Voorrips R. (2002). MapChart: software for the graphical presentation of linkage maps and QTLs. J. Heredity. 93, 77–78. doi: 10.1093/jhered/93.1.77 12011185

[B59] Wang Y. Tang H. DeBarry J. D. Tan X. Li J. Wang X. . (2012). MCScanX: A toolkit for detection and evolutionary analysis of gene synteny and collinearity. Nucleic Acids Res. 40, e49. doi: 10.1093/nar/gkr1293 22217600 PMC3326336

[B60] Wang H. Wang H. Shao H. Tang X. (2016). Recent advances in utilizing transcription factors to improve plant abiotic stress tolerance by transgenic technology. Front. Plant Science. 7, 67. doi: 10.3389/fpls.2016.00067 PMC4746321 26904044

[B61] Wang S.-M. Wang Y.-S. Su B.-Y. Zhou Y.-Y. Chang L.-F. Ma X.-Y. . (2022). Ecophysiological responses of five mangrove species (*Bruguiera gymnorrhiza*, *Rhizophora stylosa*, *Aegiceras corniculatum*, *Avicennia marina*, and *Kandelia obovata*) to chilling stress. Front. Mar. Science. 9, 846566. doi: 10.3389/fmars.2022.846566

[B62] Wei M.-Y. Li H. Zhong Y.-H. Shen Z.-J. Ma D.-N. Gao C.-H. . (2022). Transcriptomic analyses reveal the effect of nitric oxide on the lateral root development and growth of mangrove plant *Kandelia obovata* . Plant Soil. 472, 543–564. doi: 10.1007/s11104-021-05271-7

[B63] Wu M. He Z. Fung S. Cao Y. Guan D. Peng Y. . (2020). Species choice in mangrove reforestation may influence the quantity and quality of long-term carbon sequestration and storage. Sci. Total Environment. 714, 136742. doi: 10.1016/j.scitotenv.2020.136742 32018964

[B64] Wu Y. Müller M. Bai T. Yao S. Gailing O. Liu Z. (2019). Transcriptome profiling in *Camellia japonica* var. decumbens for the discovery of genes involved in chilling tolerance under cold stress. Ann. For. Res. 62, 51–68. doi: 10.15287/afr.2018.1311

[B65] Yang Z. Nie G. Feng G. Han J. Huang L. Zhang X. (2021). Genome-wide identification, characterization, and expression analysis of the NAC transcription factor family in orchardgrass (*Dactylis glomerata* L.). BMC Genomics 22, 178. doi: 10.1186/s12864-021-07485-6 33711917 PMC7953825

[B66] Yong Y. Zhang Y. Lyu Y. (2019). A stress-responsive NAC transcription factor from tiger lily (LlNAC2) interacts with LlDREB1 and LlZHFD4 and enhances various abiotic stress tolerance in *Arabidopsis* . Int. J. Mol. Sci. 20, 3225. doi: 10.3390/ijms20133225 31262062 PMC6651202

[B67] Yoo S.-D. Cho Y.-H. Sheen J. (2007). *Arabidopsis* mesophyll protoplasts: a versatile cell system for transient gene expression analysis. Nat. Protoc. 2, 1565–1572. doi: 10.1038/nprot.2007.199 17585298

[B68] Yuan C. Li C. Lu X. Zhao X. Yan C. Wang J. . (2020). Comprehensive genomic characterization of NAC transcription factor family and their response to salt and drought stress in peanut. BMC Plant Biol. 20, 454. doi: 10.1186/s12870-020-02678-9 33008287 PMC7532626

[B69] Yuan X. Wang H. Cai J. Li D. Song F. (2019). NAC transcription factors in plant immunity. Phytopathol. Res. 1, 3. doi: 10.1186/s42483-018-0008-0

[B70] Zeng Z. Lu J. Wu D. Zuo R. Li Y. Huang H. . (2020). Poly(ADP-ribose) glycohydrolase silencing-mediated H2B expression inhibits benzo(a)pyrene-induced carcinogenesis. Environ. Toxicology. 36, 291–297. doi: 10.1002/tox.23034 PMC7894510 33044785

[B71] Zhang H. Ma F. Wang X. Liu S. Saeed U. H. Hou X. . (2020). Molecular and functional characterization of *CaNAC035*, an NAC transcription factor from pepper (*Capsicum annuum* L.). Front. Plant Science. 11, 14. doi: 10.3389/fpls.2020.00014 PMC7011960 32117364

[B72] Zhuo X. Zheng T. Zhang Z. Zhang Y. Jiang L. Ahmad S. . (2018). Genome-wide analysis of the NAC transcription factor gene family reveals differential expression patterns and cold-stress responses in the woody plant *Prunus mume* . Genes. 9, 494. doi: 10.3390/genes9100494 30322087 PMC6209978

