# Supplementary material for: Comprehensive analysis of the NAC transcription factor gene family in Kandelia obovata reveals potential members related to chilling tolerance
Source: Front Plant Sci. 2022 Nov 17;13:1048822. doi: 10.3389/fpls.2022.1048822 (PMC9714628; doi:10.3389/fpls.2022.1048822)
Supplement: Supplementary file 1 [file Table_1.doc]

**Supplementary Table S1. Primer used for subcelluar localization analysis of KoNACs in the protoplasts of *A. thaliana***

| **KoNAC** | **Primer pairs** | |
| --- | --- | --- |
|  | **Forward primer (5'-3')** | **Reverse primer (5'-3')** |
| KoNAC15 | CGGGTACCATGAGTTGTGAGACGCACGC | GACTCTAGATTCTTGCTTCTTCTCACCATC |
| KoNAC27 | TCGGTACCATGATCATCACGACCTTGATTG | CGCTCTAGACTTGAAAGGATTGTTGAACTC |
| KoNAC54 | AGGGTACCATGACAAGTGATTCAAAGGAG | CCTCTAGAATAGGCAGGCCAGTAGCTGTC |
| KoNAC58 | GCAGGTACCATGAATCTCTCAATAAACGG | AGTGAATTCGTCTGATAGGTGGCACAATGG |
