# Supplementary material for: Comprehensive analysis of the NAC transcription factor gene family in Kandelia obovata reveals potential members related to chilling tolerance
Source: Front Plant Sci. 2022 Nov 17;13:1048822. doi: 10.3389/fpls.2022.1048822 (PMC9714628; doi:10.3389/fpls.2022.1048822)
Supplement: Supplementary file 2 [file Table_2.doc]

**Supplementary Table S2. Primer used for qRT-PCR analysis of *KoNACs***

| **KoNAC** | **Primer pairs** | |
| --- | --- | --- |
|  | **Forward primer (5'-3')** | **Reverse primer (5'-3')** |
| KoNAC5 | TGCGGGGGACTTGTGTTATG | AAGTCGTGAGTCTCCTTGGC |
| KoNAC8 | GACCGGATACTGGAAAGCGA | CCAGCAGACCTATCCACGTT |
| KoNAC17 | AAATGGCGCTGTACGGAGAA | TTTATCTGCCCCCGTTGCTT |
| KoNAC25 | ATTCCACCCAACCGAAGACG | CAAGTCCCAGGGTTCGTGAT |
| KoNAC30 | TGATGAAGGGGAGACAAGCG | CCACAAAACGACGCCAGATG |
| KoNAC40 | AAAGAGCACAGCAACAACGG | AGGCAACGTGAAGAAACGGT |
| KoNAC46 | GGCCGAATAGAGTTACGGGG | GTGCCTTTTCCAGCACTTCG |
| KoNAC59 | ACAAGCCGGTCTACTCACAC | GGCGAAACTCGTGCATCATC |
| KoNAC68 | AGTACCCAAATGGAGCGAGG | GGCCTCCCATTGTAGAACACA |
| 18S rRNA | GGGGCTCGAAGACGATCAGA | TTAAGCCGCAGGCTCCACTC |
