# Supplementary material for: Comprehensive analysis of the NAC transcription factor gene family in Kandelia obovata reveals potential members related to chilling tolerance
Source: Front Plant Sci. 2022 Nov 17;13:1048822. doi: 10.3389/fpls.2022.1048822 (PMC9714628; doi:10.3389/fpls.2022.1048822)
Supplement: Supplementary file 4 [file Table_4.doc]

**Supplementary Table S4. Details of 20 Motifs of KoNACs**

| **Motif** | **E-value** | **Sites** | **Width** | **Multilevel consensus sequence** |
| --- | --- | --- | --- | --- |
| 1 | 1.4e-1011 | 62 | 24 | 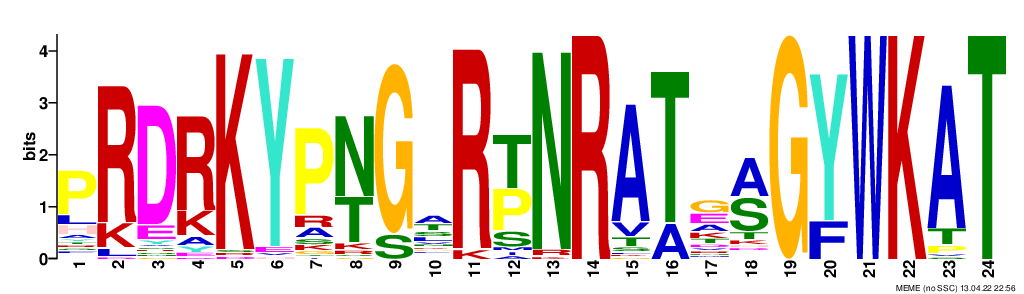 |
| 2 | 3.9e-876 | 53 | 23 | 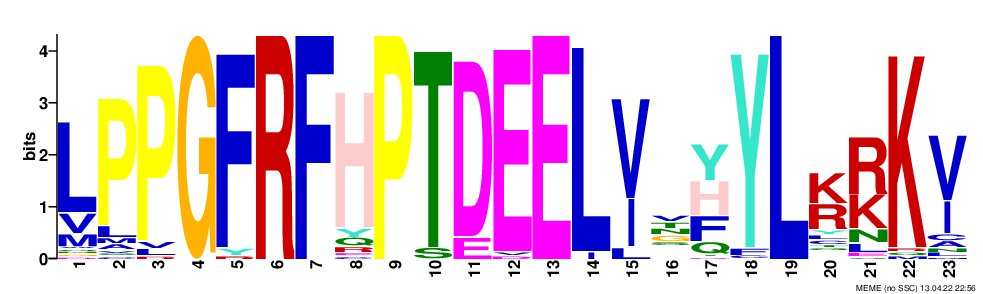 |
| 3 | 1.1e-831 | 66 | 19 | 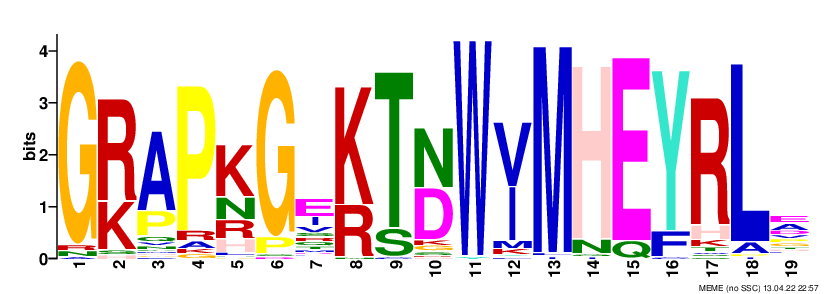 |
| 4 | 1.0e-551 | 57 | 21 | 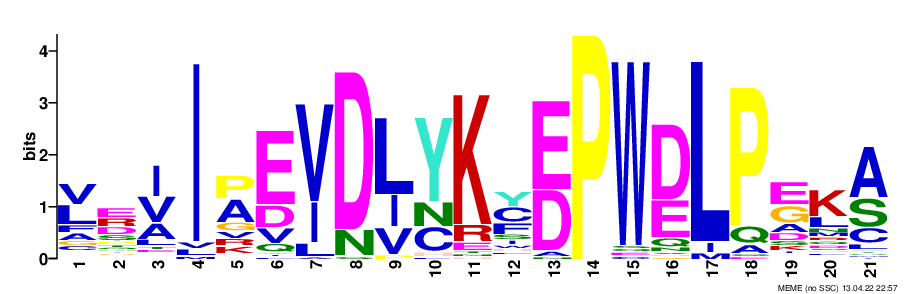 |
| 5 | 1.8e-454 | 78 | 15 | 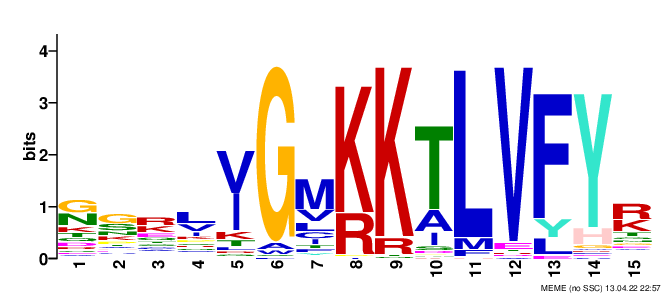 |
| 6 | 7.2e-316 | 59 | 21 | 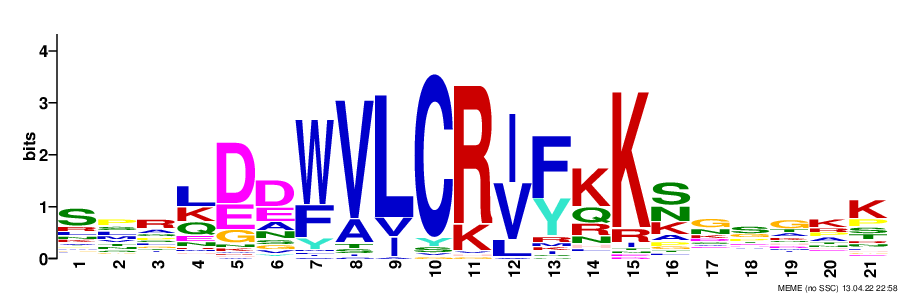 |
| 7 | 2.20E-242 | 52 | 11 | 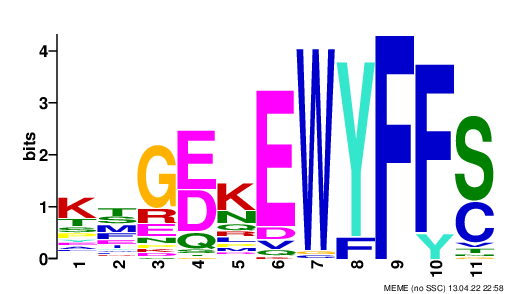 |
| 8 | 4.60E-184 | 7 | 50 | 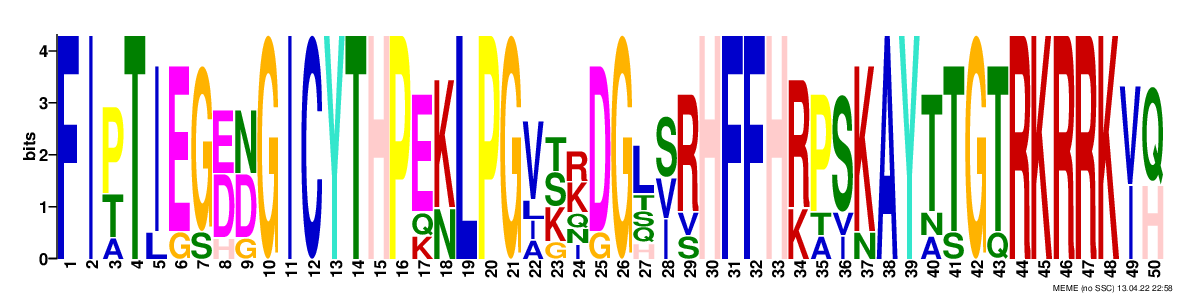 |
| 9 | 9.10E-108 | 57 | 8 | 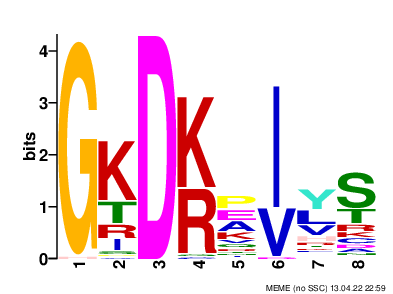 |
| 10 | 5.60E-78 | 14 | 21 | 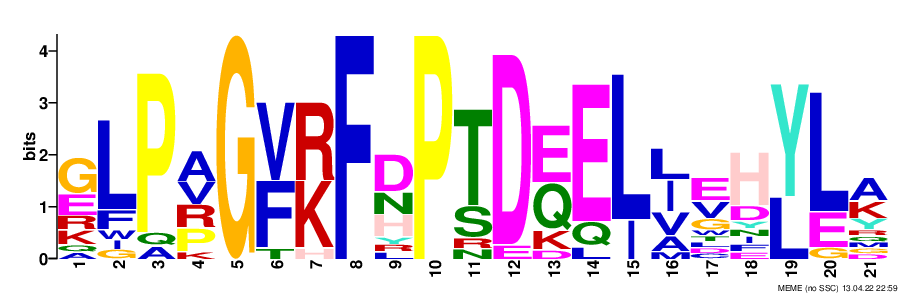 |
| 11 | 8.20E-64 | 7 | 29 | 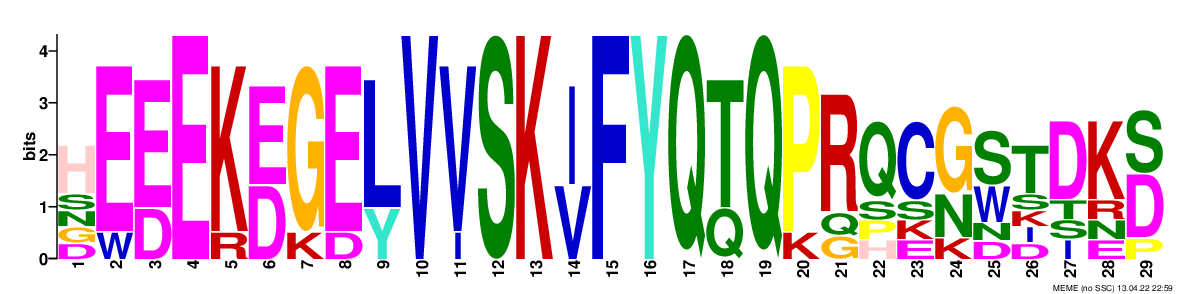 |
| 12 | 2.60E-48 | 4 | 50 | 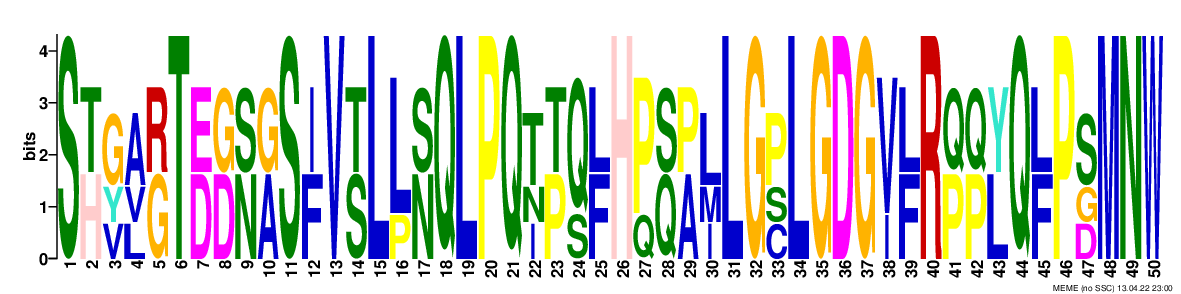 |
| 13 | 1.40E-40 | 7 | 15 | 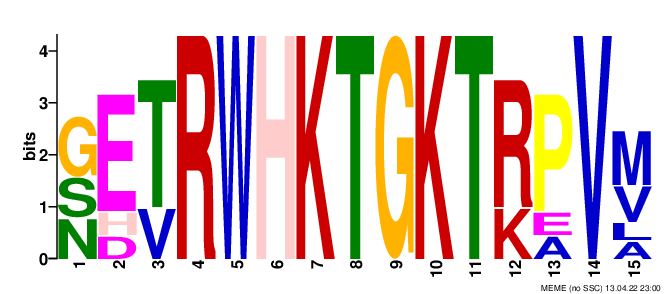 |
| 14 | 7.10E-30 | 5 | 21 | 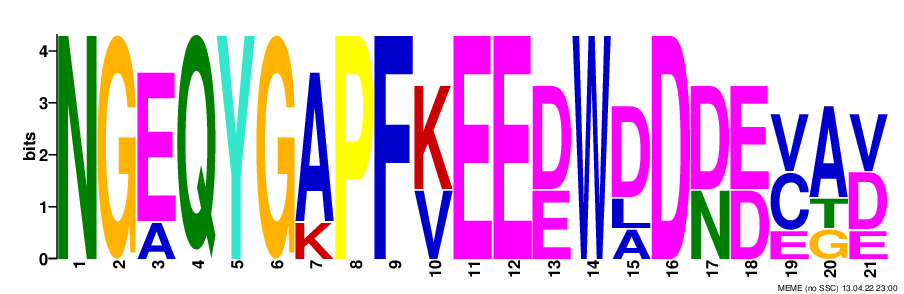 |
| 15 | 3.80E-18 | 5 | 19 | 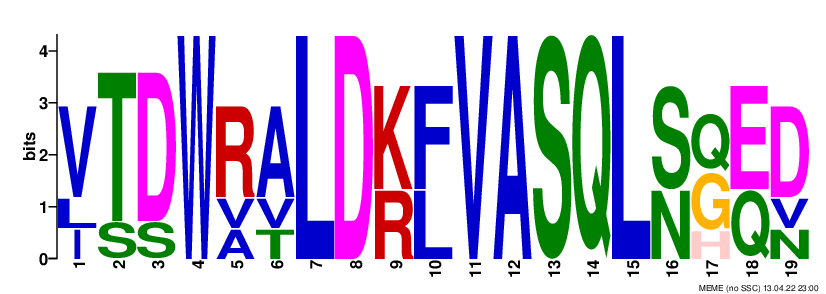 |
| 16 | 7.40E-20 | 3 | 49 | 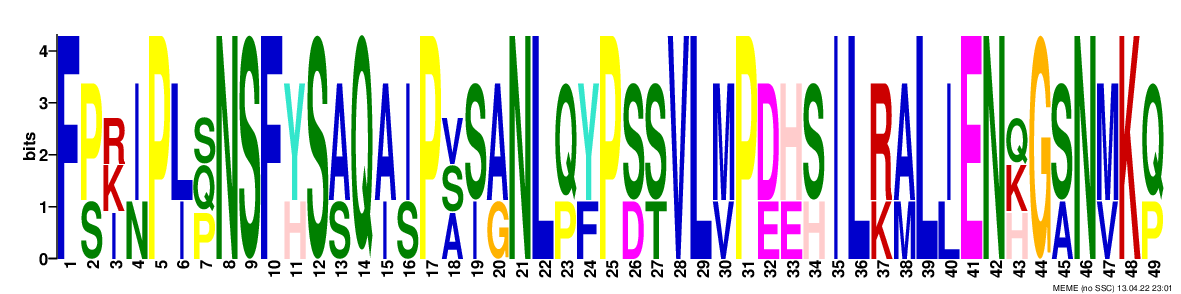 |
| 17 | 3.30E-17 | 2 | 50 | 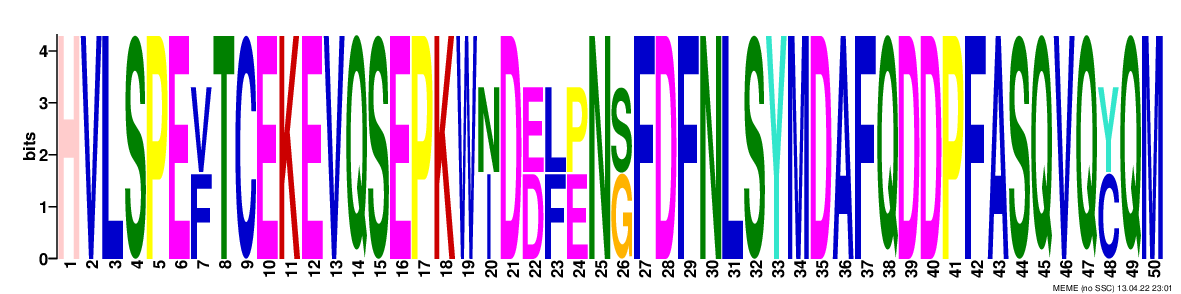 |
| 18 | 6.30E-17 | 2 | 50 | 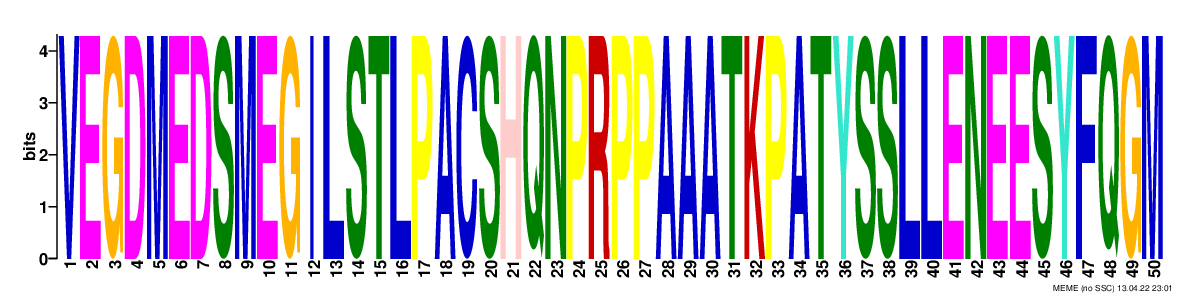 |
| 19 | 2.60E-13 | 2 | 49 | 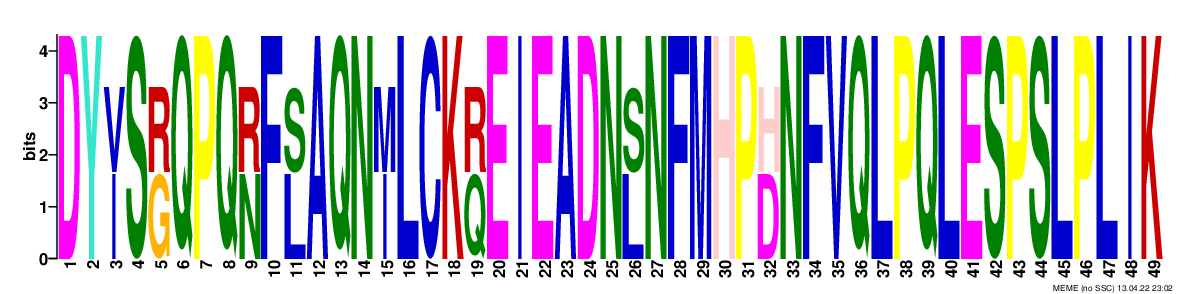 |
| 20 | 3.50E-13 | 2 | 48 | 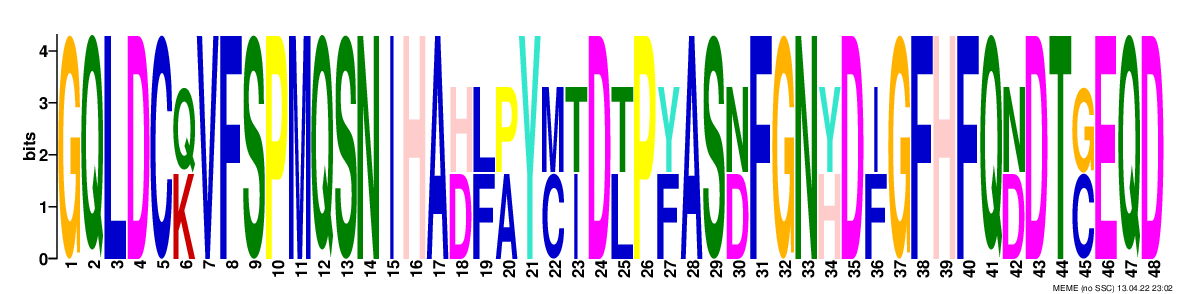 |
