# Supplementary figures and images for: Comprehensive analysis of the NAC transcription factor gene family in Kandelia obovata reveals potential members related to chilling tolerance
Source: Front Plant Sci. 2022 Nov 17;13:1048822. doi: 10.3389/fpls.2022.1048822 (PMC9714628; doi:10.3389/fpls.2022.1048822)

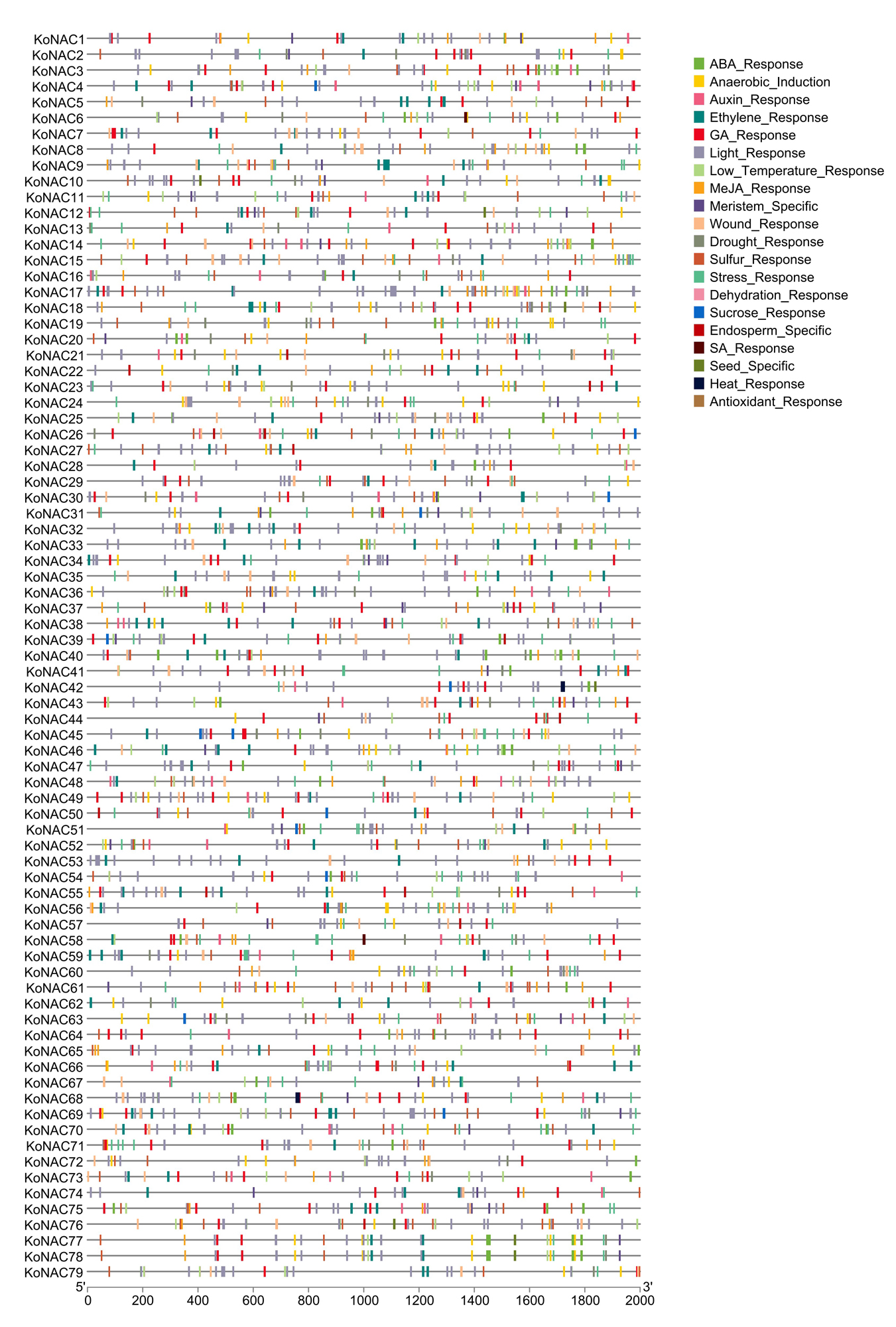

Supplement: Supplementary Figure 1 — cis-acting elements in each KoNAC. The distribution of 20 cis-acting elements in the 2000 bp upstream promoter region is shown. The different types of cis-acting elements are represented by different colors, as shown on the right. [file Image_1.jpeg]

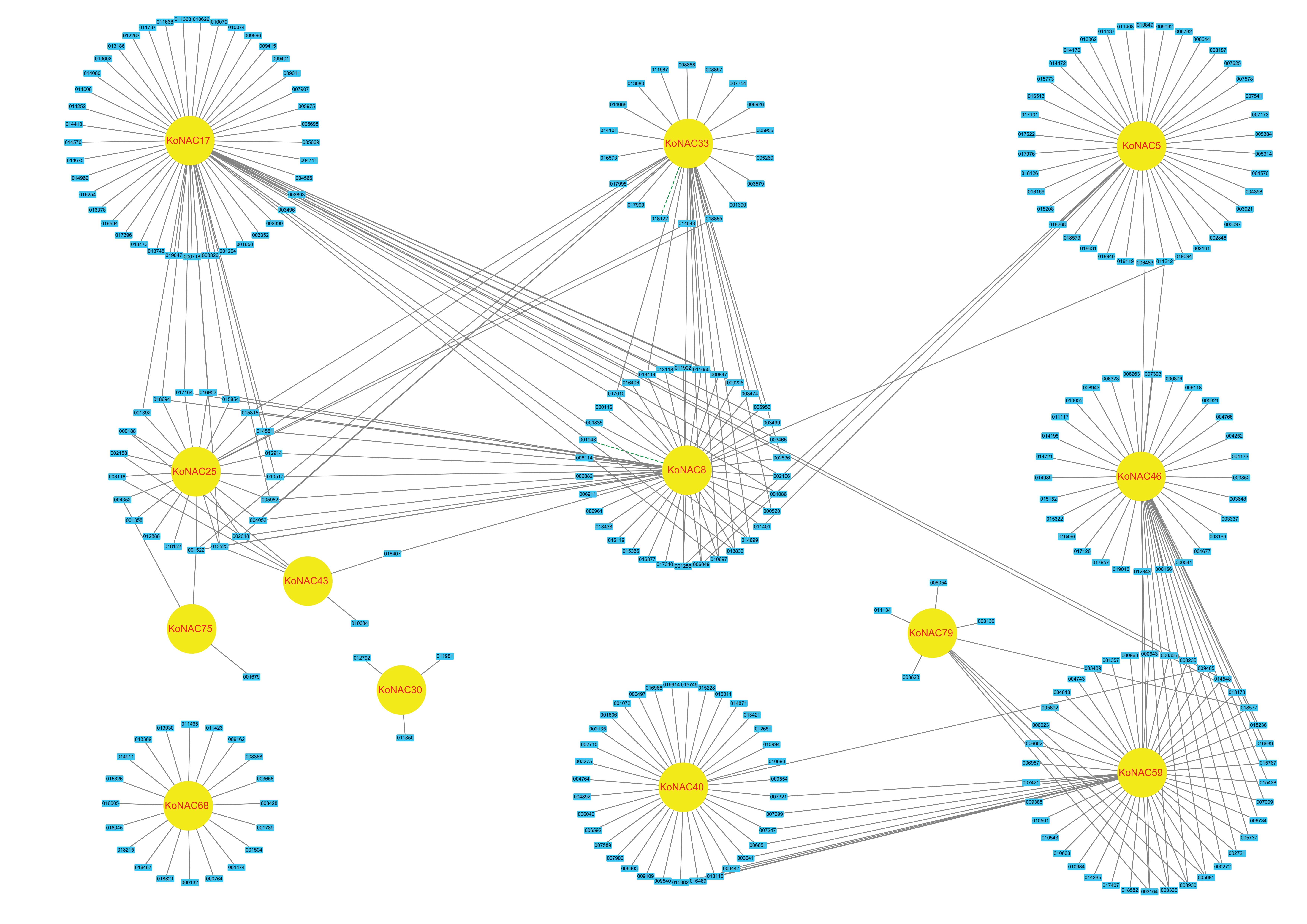

Supplement: Supplementary Figure 2 — Coexpression network of KoNACs with other genes in Kandelia obovata under chilling stress. The coexpression network was established between 13 KoNACs and 284 significantly expressed mRNAs whose absolute Pearson correlation coefficient values were equal to or greater than 0.95. Within this coexpression network, 379 and 2 pairs were positively and negatively correlated, respectively. The yellow circles represent KoNAC genes, while the blue rectangles represent the coexpressed genes. The solid gray lines represent positive correlations, while the dashed green lines represent negative correlations. [file Image_2.jpeg]

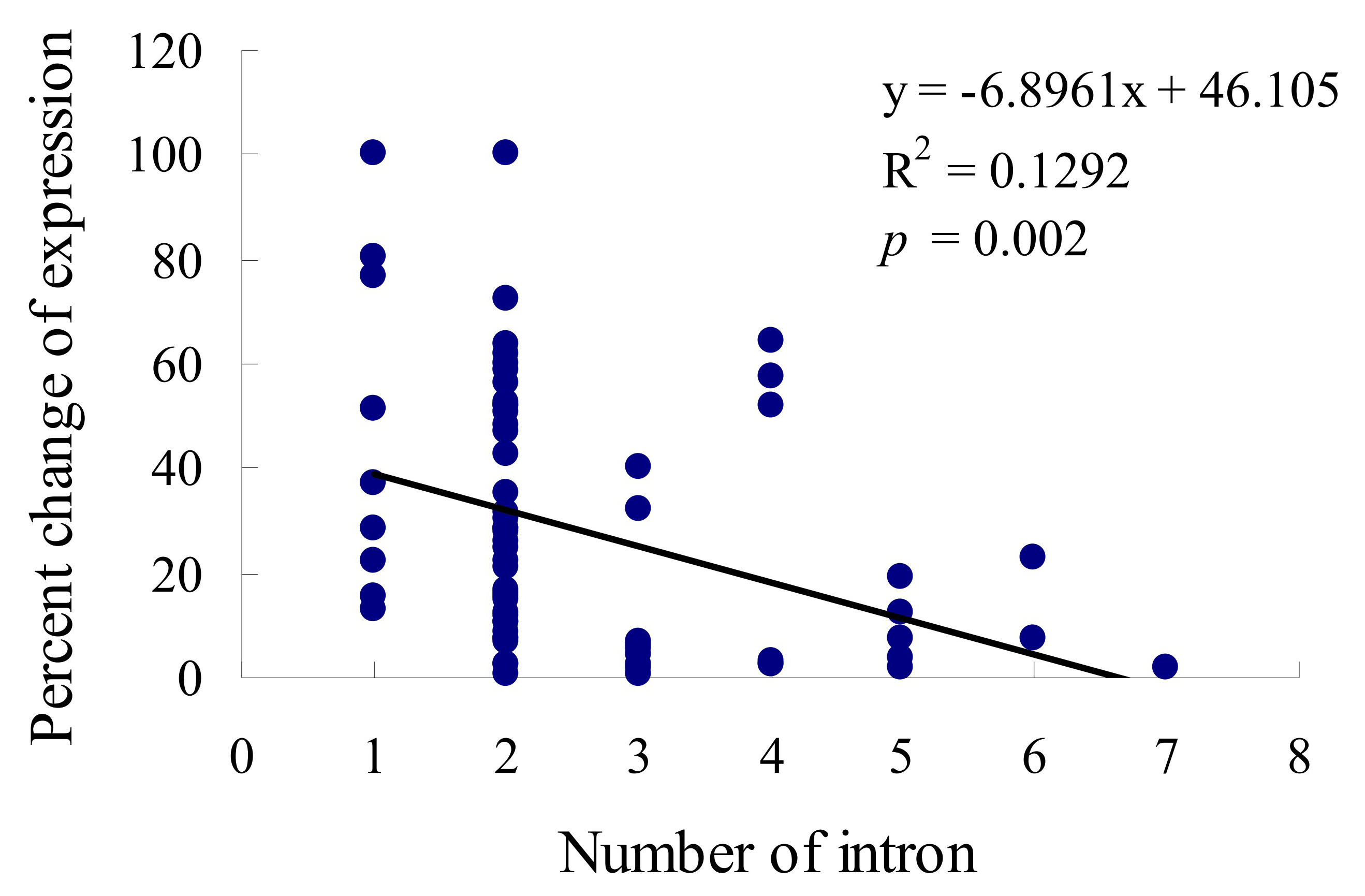

Supplement: Supplementary Figure 3 — The percent change in the expression levels of KoNACs under the first cold treatment is negatively correlated with the number of introns. The percent change in the expression levels of KoNACs under the first cold treatment was calculated with the formula (E1-E0)*100%/E0. E1 and E0 indicate the expression levels of KoNACs under the first cold treatment and in the control, respectively. [file Image_3.jpeg]
